# Developing the INCLUDE Ethnicity Framework—a tool to help trialists design trials that better reflect the communities they serve

**DOI:** 10.1186/s13063-021-05276-8

**Published:** 2021-05-10

**Authors:** Shaun Treweek, Katie Banister, Peter Bower, Seonaidh Cotton, Declan Devane, Heidi R. Gardner, Talia Isaacs, Gary Nestor, Adepeju Oshisanya, Adwoa Parker, Lynn Rochester, Irene Soulsby, Hywel Williams, Miles D. Witham

**Affiliations:** 1 Health Services Research Unit, University of Aberdeen, 3rd Floor, Health Sciences Building, Aberdeen, AB25 2ZD UK; 2 NIHR Clinical Research Network, Manchester Academic Health Science Centre, Centre for Primary Care and Health Services Research, University of Manchester, Oxford Road, Manchester, M13 9PL UK; 3 National University of Ireland Galway, School of Nursing and Midwifery, University Road, Galway, Ireland; 4 UCL Centre for Applied Linguistics, UCL Institute of Education, University College London, London, UK; 5 NIHR Clinical Research Network Cluster E, Campus for Ageing and Vitality, Newcastle University, Newcastle, NE4 5PL UK; 6 Public and Patient Representative, London, UK; 7 York Clinical Trials Unit, University of York, York, UK; 8 Translational and Clinical Research Institute; NIHR Clinical Research Network Cluster E, Campus for Ageing and Vitality, Newcastle University, Newcastle, NE4 5PL UK; 9 Public and Patient Representative, Newcastle, UK; 10 Centre of Evidence-Based Dermatology, Queen’s Medical Centre, Nottingham University Hospitals NHS Trust, Nottingham, NG7 2UH UK; 11 NIHR Newcastle Biomedical Research Centre, Campus for Ageing and Vitality, Newcastle University and Newcastle upon Tyne NHS Trust, Newcastle, NE4 5PL UK

**Keywords:** Ethnicity, External validity, Inclusion, Randomised controlled trials, Methodology

## Abstract

**Background:**

Ensuring that a trial is designed so that its participants reflect those who might benefit from the results, or be spared harms, is key to the potential benefits of the trial reaching all they should. This paper describes the process, facilitated by Trial Forge, that was used between July 2019 and October 2020 to develop the INCLUDE Ethnicity Framework, part of the wider INCLUDE initiative from the National Institute for Health Research to improve inclusion of under-served groups in clinical research studies.

**Methods:**

Development of the Framework was done in seven phases: (1) outline, (2) initial draft, (3) stakeholder meeting, (4) modify draft, (5) Stakeholder feedback, (6) applying the Framework and (7) packaging. Phases 2 and 3 were face-to-face meetings. Consultation with stakeholders was iterative, especially phases 4 to 6. Movement to the next phase was done once all or most stakeholders were comfortable with the results of the current phase. When there was a version of the Framework that could be considered final, the Framework was applied to six trials to create a set of examples (phase 6). Finally, the Framework, guidance and examples were packaged ready for dissemination (phase 7).

**Results:**

A total of 40 people from stakeholder groups including patient and public partners, clinicians, funders, academics working with various ethnic groups, trial managers and methodologists contributed to the seven phases of development. The Framework comprises two parts. The first part is a list of four key questions:

1. Who should my trial apply to?
2. Are the groups identified likely to respond in different ways?
3. Will my study intervention make it harder for some groups to engage?
4. Will the way I have designed the study make it harder for some groups to engage?

The second part is a set of worksheets to help trial teams address these questions. The Framework can be used for any stage of trial, for a healthcare intervention in any disease area. The Framework was launched on 1st October 2020 and is available open access at the Trial Forge website: https://www.trialforge.org/trial-forge-centre/include/.

**Conclusion:**

Thinking about the number of people in our trials is not enough: we need to start thinking more carefully about *who* our participants are.

**Supplementary Information:**

The online version contains supplementary material available at 10.1186/s13063-021-05276-8.

## The INCLUDE Ethnicity Framework

This paper describes the process used to develop the INCLUDE Ethnicity Framework, which was launched on 1st October 2020 (https://www.trialforge.org/trial-forge-centre/include/). The Framework was developed to help trial teams to think about the ethnicity of the people who should be involved in their trials as participants (and the trial team, especially patient and public partners), and how to facilitate their involvement. We have chosen to describe the process of how the Framework was developed, especially with regard to our external consultation, and because it might serve as a model that others could use for similar work.

**Fig. 1:**
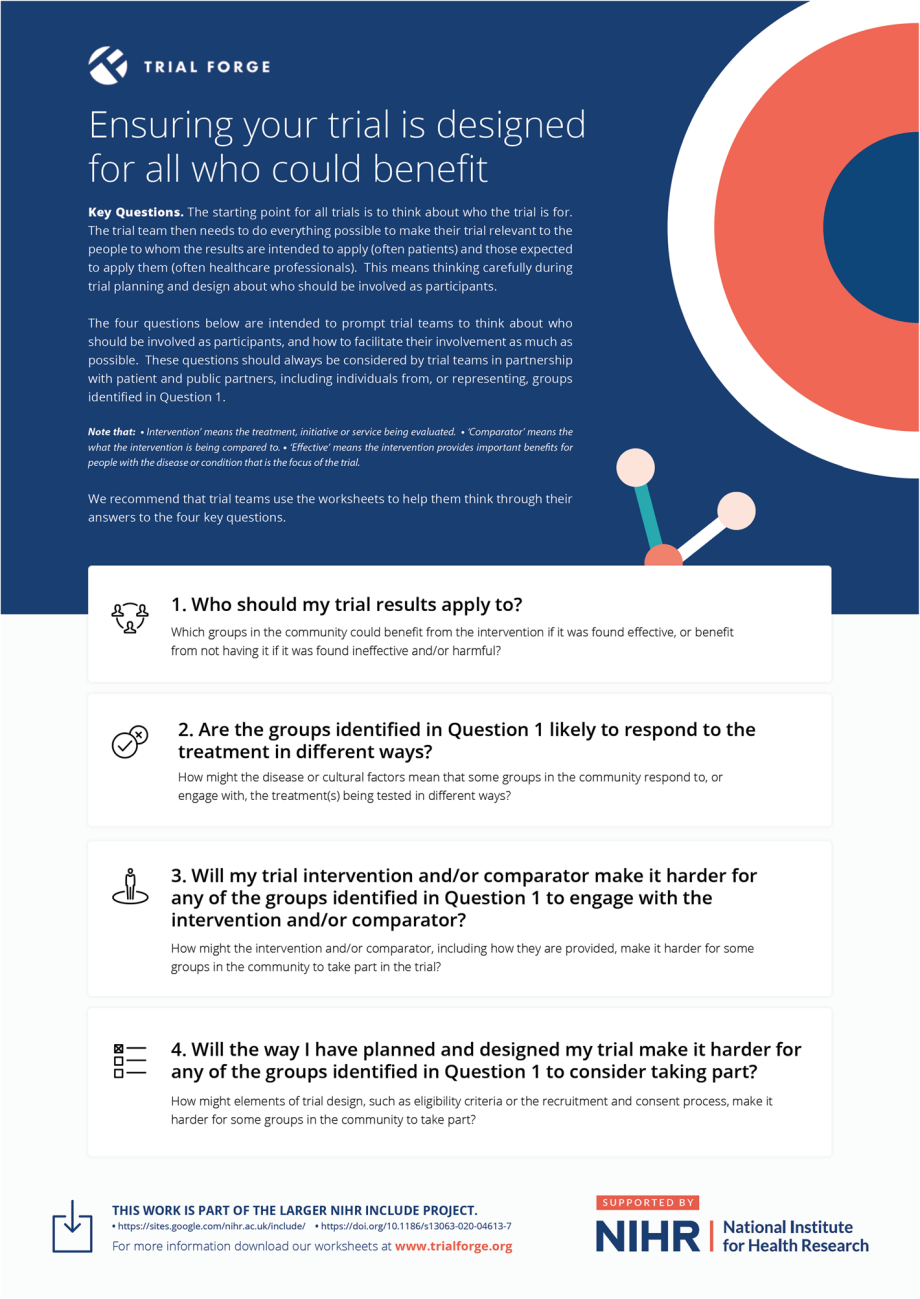
The final four key questions of the INCLUDE Ethnicity Framework

The Framework consists of four key questions and a set of worksheets to help trial teams to reflect on how to foster inclusion for the relevant groups given the context of the trial. Figure 1 shows the final four questions and the full INCLUDE Ethnicity Framework is in Supplementary File 1. The Framework is part of the broader INCLUDE initiative (https://sites.google.com/nihr.ac.uk/include/) from the National Institute of Health Research (NIHR), the UK’s biggest public funder of trials, which aims to improve inclusion of under-served groups in clinical research studies [1]. While INCLUDE is concerned with all under-served groups, the INCLUDE Ethnicity Framework focuses on identifying the ethnic groups needed for a trial and on identifying challenges to ensuring their inclusion. The Framework can be used for any stage of trial, for a healthcare intervention in any disease area and is aimed primarily at the people who design and run trials, clinicians and others who plan and design studies.

## Background

Randomised trials are important because done well they can change the health of, and the care received by, millions of people worldwide. Ensuring that a trial is designed so that the people in the trial reflect those who might benefit from the results, or be spared harms, is key to the potential benefits of the trial reaching all they should. This means thinking carefully about exactly who should participate in the trial.

This does not always happen. Narrow eligibility criteria, reliance on recruitment strategies that work for some but not all (e.g. postal invitations), assuming everyone has the same trust in health research, and a perception that some groups are more difficult or costly to recruit (e.g. due to language barriers) means trials do not always include members of the community for whom the trial intervention is a potential treatment. Examples of groups under-served by trials include pregnant people, older adults, those with multiple long-term conditions, the socioeconomically disadvantaged, or those with disabilities to name a few [1]. Ethnic minority groups are often under-represented in trials despite being 1 in 8 of the UK population and frequently having most to gain clinically [2, 3]. In a review of 12 trials for patients with type 2 diabetes, the mean percentage of South Asian recruits in eight of them was 5.5% despite South Asians representing 11.2% of the UK type 2 diabetes population [3]. The other four studies did not report ethnicity. Despite ethnic minority involvement being part of the US National Institutes of Health peer-review process for 20 years, ethnic minority individuals remain both disproportionately burdened with cancer and under-represented in cancer trials [4]. The US Food and Drug Administration issued guidance in November 2020 recommending that more inclusive trial enrolment practices should be adopted to ensure that study populations look like community populations that might use the drug if approved [5].

Not all trials need to be mirror images of the communities they serve. There may be no reason to suspect biological, social or cultural differences between those inside and outside the trial. The safety and effectiveness of the treatment might then be anticipated to be similar, although there is often neither data nor scientific understanding to support this assumption. However, there are at least four reasons why trial teams should always think carefully about who needs to be in the trial:

1. Sometimes there are treatment differences. In diabetes, there is evidence that age and genetic factors all contribute to metformin discontinuation in the absence of treatment failure, with age dominating [6]. ACE inhibitors are less effective in African Americans for hypertension [7] and some drug treatments for hepatitis C have markedly different effects on sustained virologic response across ethnic groups [8].
2. It is not just about biology. Not all treatments are based primarily on a biological response—some aim to achieve behavioural change for example—and here there will almost always be some variation in how people from different societal and cultural backgrounds respond to interventions aiming to change behaviour. The intervention may not be appropriate for some members of society to engage with; culture is known to influence care delivery to ethnic groups (e.g. how illness and its causes are perceived, or attitudes towards healthcare providers) [9]. For example, ethnic minority groups are at higher risk of mental ill health but are less likely to access mental health support in primary care and more likely to end up in crisis care, strongly suggesting that current service provision is not working for ethnic minority groups [10]. Particular ethnic groups may not be excluded explicitly but failing to actively promote their engagement with trials could mean that they are denied an effective treatment in the future or continue with a less effective or harmful treatment. An intervention offered in a place people do not visit, at a time when they cannot engage, in a language they do not understand by a person their community is unable to trust will not benefit these individuals even if it works well for others in society.
3. Relevance. If a patient asks the question ‘Do these results apply to me?’ and the research includes no one like the patient, it is hard to answer yes with any confidence. Health professionals may also be more likely to disregard the results [11].
4. Equity. Everyone should have the same chance to be included. If under-served groups are marginalised in health research, we exacerbate disengagement not only with research, but with healthcare services in their wider sense.

## Developing the INCLUDE Ethnicity Framework

In 2017, the NIHR initiated a project called ‘Innovations in Clinical Trial Design and Delivery for the under-served (INCLUDE) [1]. INCLUDE aimed to develop a roadmap to identify where research practice could improve inclusion of under-served groups, as well as provide a framework for the development of context-specific guidance to improve inclusion of particular under-served groups. The Medical Research Council (MRC) Hubs for Trials Methodology Research Recruitment and Retention Working Group (now part of the MRC-NIHR Trial Methodology Research Partnership) was at the same time starting efforts to improve representation within trials, particularly of ethnic minority individuals. The two groups and Trial Forge, an initiative to improve the efficiency of trials (https://www.trialforge.org), came together in late 2018 to develop more detailed guidance for one component of the general roadmap: ethnicity.

Work on the INCLUDE Ethnicity Framework began in earnest in July 2019. There were seven phases of work between July 2019 and Sept 2020:

1. Developing an outline of what was needed
2. Developing an initial draft of the Framework
3. Discussing that draft with a wider stakeholder group
4. Modifying the draft considering feedback from stakeholders
5. Stakeholder feedback on the modified draft
6. Applying the Framework to 3–5 trials
7. Packaging the Framework, examples and other materials

**Fig. 2:**
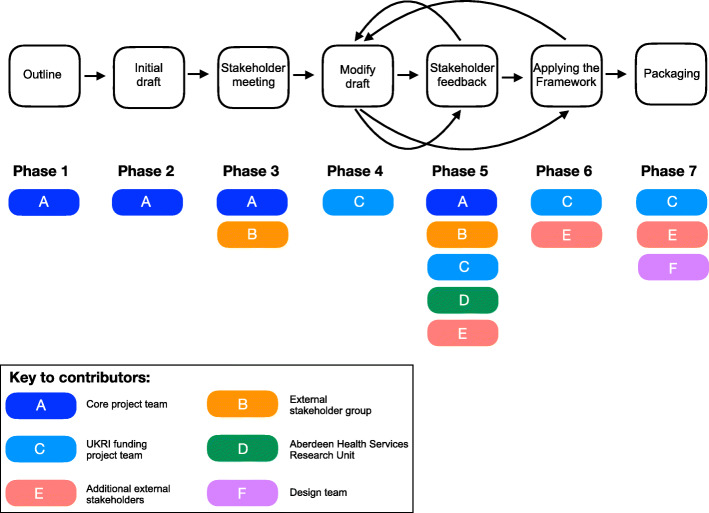
Overview of the seven development phases for the INCLUDE Ethnicity Framework and the groups contributing to each phase

A full list of the 40 individuals who participated in phases 1–7, together with the perspective each considered they brought to discussions, is available in Supplementary File 2. Most of these were involved because they were members of INCLUDE, Trial Forge or the MRC Hubs for Trials Methodology Research Recruitment and Retention Working Group. Phases 3 and 5 were different and involved more external stakeholders and details of how individuals were selected for these phases is given below. Figure 2 gives an overview of the seven phases and the groups contributing to each phase. Each phase was iterative to some extent but especially phases 4–6. The shape of iterative development was always the same: a group of individuals were invited to comment on an idea or draft, comments collected by the core project team, the idea or draft was modified and recirculated for further comment. When there were no further comments, the core project team accepted that version as agreed and moved to the next task. We did not use a formal process for this but our discussion-rich process produced plenty of comment, changes and eventual agreement. Table 1 lists the key points regarding the development of the INCLUDE Ethnicity Framework.

**Table 1:** The key points regarding the development of the INCLUDE Ethnicity Framework

Key points • The INCLUDE Ethnicity Framework is a tool that helps trial teams to think about how disease prevalence and severity, culture, faith, language, intervention and trial design features may affect trial participation of individuals from different ethnic groups. • A total of 40 people from stakeholder groups including patient and public partners, clinicians, funders, academics working with ethnic minority groups, trial managers and methodologists contributed to seven phases of Framework development. • The Framework comprises two parts: ‣ Part 1 has four key questions: 1. Who should my trial apply to? 2. Are the groups identified likely to respond in different ways? 3. Will my study intervention make it harder for some groups to engage? 4. Will the way I have designed the study make it harder for some groups to engage? ‣ Part 2 is a set of worksheets to help trial teams address these questions. • The Framework can be used for any stage of trial, for a healthcare intervention in any disease area. • The INCLUDE Ethnicity Framework was launched on October 1st 2020 and is available open access at https://www.trialforge.org/trial-forge-centre/include/. The site also contains guidance, examples of how the Framework can be applied to trials and a video giving public perspectives on why it is important for researchers to think about ethnicity at the inception of trial planning, throughout the lifespan of the trial, and/or retrospectively.	

### Phase 1: Developing an outline of what was needed (July 2019–October 2019)

A subgroup of members of INCLUDE, Trial Forge and the MRC Hubs for Trials Methodology Research Recruitment and Retention Working Group (the core project team, a total of ten individuals) discussed, mainly by email, tasks that needed to be done to support a face-to-face meeting to be held in Newcastle, UK, in October 2019. The two key conclusions from this phase were:

1. We would need a discussion document for the meeting that provided an overview of potential ways of describing ethnic groups. We recognised that we would need to be clear how we defined ethnicity for this work and that we would need to describe this. Although likely to be imperfect, we also recognised that we would need to categorise ethnic groups in some way.
2. We needed a list of trial features that potentially affect the willingness and/or ability of individuals from different ethnic groups to take part in a trial.

The documents produced in response to #1 and #2 are Supplementary File 3 and Supplementary File 4, respectively.

### Phase 2: Developing an initial draft of the Framework (October 2019–February 2020)

The two documents from phase 1 were discussed on 2nd October 2019 in Newcastle, UK, with the meeting being attended by all ten individuals who took part in Phase 1. We also discussed what the future INCLUDE Ethnicity Framework might look like and who we should invite to a wider, face-to-face stakeholder meeting. We agreed that:

1. While we wanted our future Framework to have global relevance, our choice of ethnic categories should focus on the UK because INCLUDE is a UK initiative.
2. Leading from #1, we decided to use the five main ethnic categories currently used in the UK census (Table 2). All those present were clear that categorisation of ethnicity is both challenging and sensitive and that such social categories are, by nature, imperfect. We did not feel that developing an alternative set of ethnic categories to stand in for those that are in widespread use in the UK was a helpful option for target end-users of the tool, when the groups who participate in UK trials tend to be framed using those categories, sometimes with greater granularity.
3. We considered the categories to be a minimum—in other words, trial teams would always have to consider the UK census categories when designing their trials with UK participants. However, for a given trial, this may be insufficient and attention would need to be given to specific groups within these categories. We agreed that our guidance should be clear on this point. Having the ethnic categories separate to the INCLUDE Ethnicity Framework meant that use of the Framework outside the UK would mainly be about replacing the ethnic categories with more contextually appropriate categories rather than having to modify the Framework itself.
4. We thought all the factors listed as potentially affecting the willingness and/or ability of different ethnic groups to participate in a trial (Supplementary File 3) were relevant. We discussed others too and agreed that the list should be extended prior to wider stakeholder discussion.
5. We thought the future INCLUDE Ethnicity Framework would work best as a tabulated worksheet comprising a series of questions with space for trial teams to reflect. The features discussed in #4 would be the questions in the tabulated worksheet. We also recognised that funders are key to the effective implementation of the future Framework. We thought of this in the same way as Public and Patient Involvement, which became widespread in the UK and elsewhere once major funders required it. The first draft of the Framework is Supplementary File 5; this version was used in Phase 3.
6. We agreed an initial list of potential invitees for the future stakeholder meeting, which we aimed to hold in early 2020.

**Table 2:** The ethnic categories used by the UK census

Broad category	Sub-categories
White	• English/Welsh/Scottish/Northern Irish/British • Irish • Romani or Irish Traveller • Any other White background
Mixed/multiple ethnic groups	• White and Black Caribbean • White and Black African • White and Asian • Any other mixed/multiple ethnic background
Asian/Asian British	• Indian • Pakistani • Bangladeshi • Chinese • Any other Asian background
Black/African/Caribbean/Black British	• African • Caribbean • Any other Black/African/Caribbean background
Other ethnic group	• Arab • Any other ethnic group

### Phase 3: Discussing the initial draft with a wider stakeholder group (February 2020–March 2020)

A face-to-face meeting with stakeholders was held in London, UK, on 4th February 2020. The meeting had 17 participants of whom six were patient and public representatives. The latter included the patient and public representative from the core project team, two individuals identified through other ethnicity work being done in Aberdeen and three individuals identified through www.peopleinresearch.org, NIHR website to identify members of the public interested in contributing to research design. Five of the six patient and public representatives brought a perspective that they considered to Black British, Black African, Black-African-British, Black British-Ghanaian, or British South Asian. One participant did not list ethnicity in his perspective and one participant preferred not to be named or to state a perspective. In addition to representation from patients and public, the core project team thought the meeting needed representation from clinicians, academics with experience of working with ethnic minorities, funders, trial methodologists, linguists, individuals with experience in facilitating public involvement in setting research agendas, research design support services and ethics committees. We used our personal contacts to identify potential individuals and were successful in involving stakeholders from all groups except research design support services. Our ethics committee representative could not attend on the day but did contribute afterwards.

After a brief presentation on the INCLUDE initiative, participants had an open plenary discussion on the draft Ethnicity Framework (Supplementary File 5), which focused on first impressions and thoughts. Two small group discussions later in the day asked more specific questions:

1. *Group work #1 –* We came up with three key design considerations: (1) characteristics of a particular ethnic group that might influence the effect of treatment, (2) the intervention and (3) trial design and delivery. We asked small groups to consider whether there was something missing in this list, or whether something needed to be changed.
2. *Group work #2 –* Given Group work #1, what can we give to trialists to help them to achieve more appropriate ethnic diversity in their trial populations? We want something that will achieve change and is practical.

These discussions were wide-ranging and among other things covered use of the term ‘under-served’ rather than alternatives. Discussions emphasised the importance of trial teams asking the question ‘For whom will the trial results be important?’ and acknowledged the challenges of categorising ethnicity, including for reasons of individual and group identity. We also underscored the importance of trial teams reflecting on the impact culture and beliefs may have on trial engagement, highlighted other ongoing work and tools broadly focusing on ethnicity and health and underlined that trial teams need to build trust and engage more with their communities if under-served populations are to be better served. The full summary from the meeting is Supplementary File 6.

A key suggestion was to get trial teams to think more about the composition of their target and trial population at the design stage by asking them four key questions:

1. Who should my trial apply to?
2. Are the groups identified likely to respond in different ways?
3. Will my study intervention make it harder for some groups to engage?
4. Will the way I have designed the study make it harder for some groups to engage?

Originally, we had anticipated that these questions would be accompanied by examples, but it was suggested that too much ‘spoon-feeding’ would encourage a checklist approach rather than encourage teams to reflect and think more deeply about the populations they serve. A key implementation decision was therefore to create a form with just the main questions followed by blank spaces for teams to write in to stimulate such thought, with examples provided elsewhere to help with understanding of the questions and how ethnicity might interface with the questions posed.

This suggestion captured much of the discussion in a concrete and actionable way. We also agreed some principles for future work:

- The purpose of this work is to better serve ethnic minority groups in health research, especially within the National Health Service (NHS).
- The target audience for our materials is primarily those who design and deliver trials.
- The target behaviour to support is the routine consideration of ethnicity.
- We need to consider what success looks like, which implies that it can be measured.

The main action coming from the meeting was to embed the four key questions into a new draft of the Framework, while bearing in mind the principles and other suggestions noted in the meeting summary. This included the need for a small example set of trials to which the Framework had been applied.

### Phase 4: Modifying the draft considering feedback from stakeholders (June 2020–July 2020)

Work on the Framework was delayed by the outbreak of the COVID-19 pandemic, which took hold in the UK in early March 2020. Work re-started in June 2020 with funding received from the UK Research & Innovation (UKRI)–NIHR COVID-19 Rapid Response call linked to ethnicity.

The meeting summary from phase 3 was used as the basis for a new version of the Framework (Supplementary File 7). All nine of those listed as contributing to phase 4 in Supplementary File 1 then made iterative changes to the draft Framework based on email and video-conference discussions.

We decided to make the four key questions relevant to all under-served groups but make the tabulated worksheets focus specifically on ethnicity. By keeping the four key questions general, other under-served groups are not forgotten while at the same time the ethnicity-focused worksheets could become a model for similar worksheets in the future for other under-served groups. The final modified draft from phase 4 that went into the phase 5 consultation is Supplementary File 8.

### Phase 5: Stakeholder feedback on the modified draft (July 2020–August 2020)

Consultation on the draft Framework from phase 4 began as soon as the draft was available and a total of 31 individuals were involved, including all who attended the February 4th meeting. The core project team also involved an additional academic with experience of working with ethnic minority groups and the founder of a patient engagement agency. Comments mainly came by email, but we also had some videoconferences and an internal seminar at the Health Services Research Unit, University of Aberdeen, where the draft Framework was presented to research staff who had previously had no involvement with the work.

The response was overwhelmingly positive but there were many suggestions for improvements, especially to the worksheets. Changes made in light of feedback included:

- The addition of more explanatory text to go with the four key questions and the worksheets. This text explained the purpose of the questions and worksheets, that patient and public partners need to be involved and that there are potential differences within ethnic groups, i.e. that ethnic groups are not homogenous.
- Dropping the word ‘biological’ from the worksheets in favour of ‘disease’. There was inconsistency in our use of these words and we favoured the use of the term ‘disease’.
- Adding text to make clear which of the key questions a particular worksheet helped the trial team to address.
- Adding a new question to the worksheets to ask whether people in each ethnic group might access healthcare differently.
- Adding a new question to the worksheets to ask whether the trial outcomes themselves, or other data being collected, might limit participation.
- Adding a new reference in the worksheets to video calls as an example mode of delivery for the intervention or comparator.
- Made substantial presentational changes to the tables, for example giving each question its own row in the table and to number the worksheets for ease of reference.
- Numerous minor text changes and typographical corrections.

By late July 2020, we had a version of the Framework that we could begin use in phase 6 while additional feedback was still coming in. The late August version of the Framework used for most of the phase 6 work is Supplementary File 9.

### Phase 6: Applying the Framework to 3-5 trials (July 2020 - Aug 2020)

One of the suggestions coming from the February 2020 meeting was that we should prepare a small set of examples of real trials to which the Framework had been applied. The nature of the UKRI-NIHR COVID-19 funding we received meant that at least some of these had to be COVID-19 trials. We also wanted to include a type 2 diabetes trial because we already knew prevalence is higher in some ethnic groups than others, and we wanted a mix of intervention types and settings. We selected the following six trials:

1. By Band Sleeve – a UK bariatric surgery trial (http://www.isrctn.com/ISRCTN00786323).
2. COVAC1 – a UK trial evaluating a potential vaccine for COVID-19 (http://www.isrctn.com/ISRCTN17072692).
3. iQuaD – a UK community-based periodontal disease prevention trial (http://www.isrctn.com/ISRCTN56465715).
4. PRINCIPLE – a UK trial for community-based treatment of COVID-19 (http://www.isrctn.com/ISRCTN86534580).
5. RECOVERY – a UK platform trial evaluating treatments for COVID-19 (http://www.isrctn.com/ISRCTN50189673).
6. TriMaster – a UK type 2 diabetes treatment trial (http://www.isrctn.com/ISRCTN12039221).

The Aberdeen-based team divided these trials between them so that the Framework was applied to each trial independently by two people. The exception was RECOVERY, which was the first trial to which we applied the Framework and all four of the Aberdeen team independently applied the Framework to this trial. Consensus was then reached between the pairs of assessors (or four for RECOVERY) on the final completed Framework for each trial.

We had no pre-formulated method for answering the worksheet questions, but the approach taken by all assessors was the same: web-based searching. This was not formal, systematic searching (as done for a systematic review) but more ad hoc, looking for information that could shed some light on potential challenges and issues. We recognised that retrospectively applying the Framework to trials with which we had little or no involvement (iQuaD was the only trial where there was Aberdeen involvement) was not the way in which the Framework is intended to be used. The information in our example worksheets may therefore not be a proper reflection of the trial because we did not have in-depth knowledge of the field, or access to all the trial materials. We added a statement making this point to the beginning of each example, together with the names of the assessors and the web-links to the main sources of information about the trial that we used (namely trial registration documents, the protocol and study website if there was one). We discussed the way we answered the worksheet questions with three of our patient and public partners and two independent researchers with experience of working with ethnic minority groups. Their feedback was overwhelmingly positive. Prior to completing the examples, all six were checked for consistency with each other in terms of language and presentation.

Table 3 gives examples of the sort of challenges to diverse ethnic group involvement that using the Framework helps trial teams to identify. These are all taken from the six trials listed above and the fully completed Frameworks for these trials are available at https://www.trialforge.org/trial-forge-centre/include/. We will upload more examples as they become available.

**Table 3:**
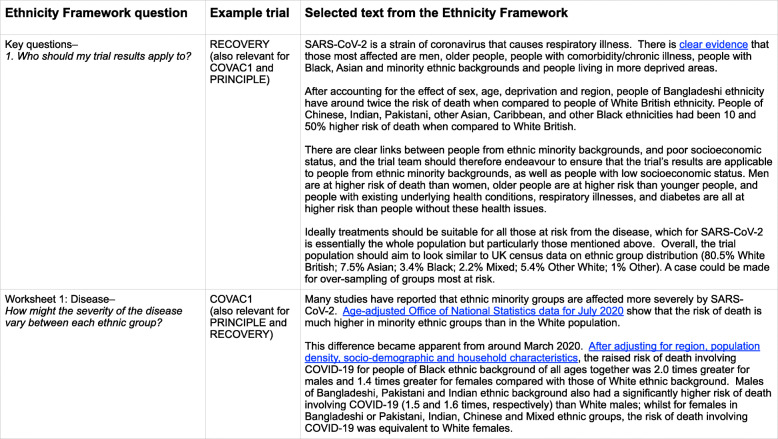
Example text taken from the INCLUDE Ethnicity Frameworks for the By-Band-Sleeve, COVAC1, iQuaD, PRINCIPLE, RECOVERY and TriMaster trials. The fully completed Frameworks for all six trials are available at https://www.trialforge.org/trial-forge-centre/include/. As we note in the Frameworks themselves, the information may not be a proper reflection of the trial because we did not have in-depth knowledge of the field or access to all the trial materials

### Phase 7: Packaging the Framework, examples and other materials (August 2020–September 2020)

The final stage of our work was to package the INCLUDE Ethnicity Framework together with some brief guidance, the examples and other materials to support the use of the Framework. This packaging work involved a design company as well as video staff at the Centre for Black and Minority Ethnic Health at the University of Leicester. The resources include a video giving public perspectives as to why thinking more carefully about ethnicity when designing trials is important. We also provide some resources to help trial teams address any participation challenges highlighted by using the Framework.

All of our materials are available at https://www.trialforge.org/trial-forge-centre/include/.

## Discussion

COVID-19 has shone a very bright, and very harsh, light on researchers’ failure to routinely consider ethnicity when designing trials [12, 13]. But while the light may be relatively new, the problem is not. The INCLUDE Ethnicity Framework aims to help trial teams to systematically consider who needs to be involved in their trial and which elements of the intervention and the trial design may present challenges for that involvement. Without this, the trial results are unlikely to be relevant to the care of all those who could benefit—inclusion is certainly an issue of moral and social justice but it is also an issue of science.

The Framework is new, imperfect, and does not offer solutions to all of the challenges it may highlight. Using it does not guarantee that trialists will get the right ethnic representation, or that inequalities will be corrected. But reflecting on the questions the Framework asks will make trial teams think more about the communities they serve and move us in the right direction.

### Potential downsides to using the INCLUDE Ethnicity Framework

We believe there are clear potential benefits to using the Framework, but there are also some potential negatives, and these are worth mentioning.

Firstly, there is a danger that the Framework could lead to quotas. This is not our intention. Careful use of the Framework should help trial teams to identify the mix of ethnic groups needed for their trial, and this will vary from trial to trial. Secondly, using the Framework will increase the workload for trial teams. The process of completing the Framework requires a modest amount of work, but the work of engaging with potential participants, and implementing changes to alleviate the barriers that the completed Framework highlights are potentially significant. It is likely that new or additional trial processes will be needed to engage a wider range of ethnic groups and there is no shying away from the fact that this will add both work and expense. However, using the Framework when developing grants should help to provide a justification to funders for that work and expense. Thirdly, it is quite possible that a trial team genuinely aiming for a diverse population will recruit more slowly than a trial team that pays less attention to who is in the trial. Changing this needs a larger set of evidence-informed resources for engaging different ethnic groups than is available at present. It also needs funders to take a broad view of trial costs. Time and money invested in trials recruiting diverse populations are likely to deliver more relevant research, with a consequent reduction in research waste and misallocated healthcare resources in the longer term. Finally, the Framework is new and there is no clear ‘best’ way to use it. This will lead to inconsistency in how it is used; it may also lead to frustration. We use ethnicity categories but, of course, individuals within ethnic groups are not homogenous; there is a danger that some Framework users may rely on stereotyping (e.g. linked to language skills, or faith-based beliefs) rather that speaking to people from the ethnic groups concerned.

We do not think that the above are sufficient justification for not using the Framework. However, we do think is worth being aware of these potential negatives, if only to make sure that counter-arguments are built into trial design proposals. We welcome suggestions for how the Framework can be improved, descriptions of how trial teams used it and how trial design decisions were influenced by its use. Any feedback can be built into future versions of the Framework and, potentially, influence similar work for other under-served groups. Please send comments to info@trialforge.org.

### The future

As with Patient and Public Involvement and Engagement in trials, funders have a key role to play in the adoption of INCLUDE and the INCLUDE Ethnicity Framework. Funders can help by signposting applicants towards these materials in their guidance for applicants. Better still would be to also make it clear to applicants that consideration of equity, diversity and inclusion will be part of the review process, as both NIHR and the Wellcome Trust already do. We consider such moves to be a sign of success for the mission of INCLUDE and the Ethnicity Framework to promote inclusion, more relevant research and a more equitable society.

Although our work has had a UK and Ireland focus, we think the questions the Framework asks are likely to be relevant elsewhere. Indeed both INCLUDE and the Framework are already being highlighted outside the UK, Belgium’s KCE Trials for example includes them in its resources for trialists (https://kce.fgov.be/en/useful-links-resources-for-investigators). In our discussions of the Framework with colleagues outside the UK and Ireland, the key challenge for international use is not whether the Framework questions are relevant but how to tailor the discussion of ethnicity to the country. Ethnicity is a complex and sensitive topic and there are sometimes substantial differences in how countries approach it, as we found in our work (see Supplementary File 3). This is why we refer to ‘ethnic groups’ in the Ethnicity Framework itself rather than to specific ethnic groups or a particular system of categorisation.

We think that the main modification for a non-UK or Ireland version of the Ethnicity Framework is to develop a country-specific version of our Appendix 1 ‘Ethnic categories’ document at https://www.trialforge.org/trial-forge-centre/include/, with more minor changes needed to the Framework itself depending on the language of ethnicity in the country of interest. We have yet to do this, but we would be very interested in hearing from groups outside the UK and Ireland who would like to try.

Our Framework focuses on ethnicity but the wider INCLUDE initiative [1, 14] has a much broader remit, acknowledging that there are many groups in society that have been forgotten, ignored and under-served by health research. The INCLUDE Ethnicity Framework provides a model for how trialists might consider some of these other groups. The MRC-NIHR Trial Methodology Research Partnership’s Inclusivity Subgroup is beginning to do this for people who are socio-economically disadvantaged, have cognitive impairments or identify as LGBTQIA+. The Framework could likewise be adapted for other under-served groups or to consider under-served groups from multiple categories (including those stated above) more holistically. The NIHR also has a detailed workplan on improving equality, diversity and inclusivity (https://www.nihr.ac.uk/about-us/our-contribution-to-research/equality-diversity-and-inclusion.htm) for participants in research and its workforce which will hopefully benefit from the INCLUDE guidance. The Ethnicity Framework’s four key questions are already general enough to cover all groups. It is, however, likely that specific differences will remain and trial designers will need to think carefully about who in the community their trial is trying to help.

Finally, the impact of the INCLUDE Ethnicity Framework on the inclusion of different ethnic groups in trials needs to be assessed and this needs funders and research networks to collect data from trial teams. There is little point to the Framework if we do not know whether it has any impact. The UK’s research infrastructure contains some strengths in this regard [15] but there remain challenges, not least the need to more routinely record data on ethnicity. How to go about recording ethnicity is not trivial. Those asking questions need to know why they are doing it and those being asked need to know how their ethnicity information will help to reduce inequalities [16]. The data need to be reported. At present we would encourage trial teams to record and report ethnicity information for their trials, but we would not mandate it until it is clearer how to do this well. Some of us are starting work in this area and we are optimistic that guidance and tools will be available in the near future.

## Conclusion

Thinking about the number of people in our trials is not enough: we need to start thinking more carefully about *who* our participants are.

## Supplementary Information

**Additional file 1.**

**Additional file 2.**

**Additional file 3.**

**Additional file 4.**

**Additional file 5.**

**Additional file 6.**

**Additional file 7.**

**Additional file 8.**

**Additional file 9.**

## Data Availability

All our data and materials are available as supplements to this article or at https://www.trialforge.org/trial-forge-centre/include/.

## Abbreviations

ACE: Angiotensin-converting enzyme
COVID: Coronavirus
INCLUDE: Innovations in Clinical Trial Design and Delivery for the under-served
LGBTQIA+: The Lesbian, Gay, Bisexual, Pansexual, Transgender, Genderqueer, Queer, Intersexed, Agender, Asexual, and Ally community
MRC: Medical Research Council
NHS: National Health Service
NIHR: National Institute for Health Research
UK: United Kingdom
UKRI: United Kingdom Research and Innovation
