# Supplementary material for: Developing the INCLUDE Ethnicity Framework—a tool to help trialists design trials that better reflect the communities they serve
Source: Trials. 2021 May 10;22:337. doi: 10.1186/s13063-021-05276-8 (PMC8108025; doi:10.1186/s13063-021-05276-8)

Ensuring your trial is designed for all who could benefit

Trial teams need to do everything possible to make their trial relevant to the people to whom the results are intended to apply (often patients) and those expected to apply them (often healthcare professionals). The four questions below are intended to prompt trial teams to think about who should be involved as participants, and how to facilitate their involvement as much as possible. These questions should be considered by trial teams in partnership with patient and public partners, including individuals from, or representing, groups identified in Question 1. Note that:

- *‘Intervention*’ means the treatment, initiative or service being evaluated.
- ‘*Comparator*’ means the what the intervention is being compared to.
- ‘*Effective*’ means the intervention provides important benefits for people with the disease or condition that is the focus of the trial.

We recommend that trial teams use the worksheets to help them think through their answers to the four key questions.

**1.** Who should my trial results apply to?

Which groups in the community could benefit from the intervention if it was found effective, or benefit from not having it if it was found ineffective and/or harmful?

**2.** Are the groups identified in Question 1 likely to respond to the treatment in different ways?

How might the disease or cultural factors mean that some groups in the community respond to, or engage with, the treatment(s) being tested in different ways?

**3.** Will my trial intervention and/or comparator make it harder for any of the groups identified in Question 1 to engage with the intervention and/or comparator?

How might the intervention and/or comparator, including how they are provided, make it harder for some groups in the community to take part in the trial?

**4.** Will the way I have planned and designed my trial make it harder for any of the groups identified in Question 1 to consider taking part?

How might elements of trial design, such as eligibility criteria or the recruitment and consent process, make it harder for some groups in the community to take part?

| **1. Who should my trial results apply to?** |
| --- |
|  |

| **2. Are the groups identified in Question 1 likely to respond to the treatment in different ways?** [**( VIEW WORKSHEET )**](#WorksheetONE) |
| --- |
|  |

| **3. Will my trial intervention and/or comparator make it harder for any of the groups identified in Question 1 to engage with the intervention and/or comparator?** [**( VIEW WORKSHEET )**](#WorksheetTWO) | |
| --- | --- |
|  | |
| 1. **Will the way I have planned and designed my trial make it harder for any of the groups identified in Question 1 to consider taking part?** [**( VIEW WORKSHEET )**](#WorksheetTHREEA) |  |
|  |  |

Worksheets for thinking through factors that might affect ethnic group involvement in a trial

These worksheets are intended to be used by trial teams in partnership with patient and public partners to ensure that ethnic group involvement is considered at the trial design stage. Before completing the worksheets, the trial team **should have answered Question 1** **of the INCLUDE Key Questions with regard to ethnic group involvement**.

The worksheet may cover issues that some trial teams already think about. The intention is that the worksheet will help to highlight issues consistently across trials for all trial teams, as well as raising some questions that may not be routinely considered at present.

Finally, while the worksheet asks trial teams to think about possible differences between ethnic groups, it is important to remember that there are also differences *within* ethnic groups, especially between generations and between men and women. No ethnic group is homogenous. See [Appendix 1](https://www.trialforge.org/trial-forge-centre/include/) for more on our definition of ethnicity.

**Worksheet 1**

This worksheet provides some questions **to guide your thinking about ethnic group involvement when answering Question 2** of the INCLUDE Key Questions.

**Disease and cultural factors that might influence the effect of treatment for some ethnic groups**

| **Disease** | How might the prevalence of the disease vary between each ethnic group in the target population? | Response: |  |
| --- | --- | --- | --- |
|  | How might the severity of the disease vary between each ethnic group? | Response: |  |
|  | How might the disease present in people from each ethnic group (this may include symptoms, type or pattern or rate of disease progression)? | Response: | |
|  | How close is the match between each ethnic group living with the disease and the ethnic groups living in the areas where the trial is to be run? | Response: | |
|  | Other factors to consider: | | |
| **Cultural** | How might perceptions of the disease and social stigma around it be different for each ethnic group in the target population? | Response: | |
|  | How might ways of describing the disease be different for each ethnic group? | Response: | |
|  | How might cultural practices, beliefs and traditions influence the acceptability of, and adherence to, the treatment(s) for each ethnic group? | Response: | |
|  | How or when might people in each ethnic group access healthcare for this disease differently? | Response: | |
|  | Other factors to consider: | | |

**Worksheet 2**

This this worksheet provides some questions **to guide your thinking about ethnic group involvement when answering Question 3** of the INCLUDE Key Questions.

**Intervention and comparator factors that might affect how some groups engage with the intervention and/or comparator***

| **What** | How might the intervention(s) and comparator limit participation of people from each ethnic group in the target population? | Response: |
| --- | --- | --- |
|  | How, and in what way, were people from each ethnic group involved in selecting or designing the trial intervention/comparator? | Response: |
|  | Other factors to consider: | |
| **Who** | How might the person delivering the intervention/comparator limit participation of people from each ethnic group in the target population? | Response: |
|  | Other factors to consider: | |
| **How** | How might the mode of delivery (e.g. telephone, video-call, face-to-face, in groups) limit participation of people from each of the ethnic groups in the target population? | Response: |
|  | Other factors to consider: | |
| **Where** | How might where the intervention/comparator is delivered (e.g. hospital, general practice, local library) limit the participation of people from each ethnic group in the target population? | Response: |
|  | Other factors to consider: | |
| **When & Intensity** | How might when the intervention/comparator is delivered (e.g. during working hours) or the intensity (e.g. number of times it is delivered, over what period, time commitment for each session and overall) limit participation of people from each ethnic group in the target population? | Response: |
|  | Other factors to consider: | |

*These factors are taken from TIDieR ([http://www.equator-network.org/reporting-guidelines/tidier/](about:blank)).

**Worksheet 3a**

This worksheet provides some questions **to guide your thinking about ethnic group involvement when answering Question 4** of the INCLUDE Key Questions.

**Trial eligibility and participation factors that might affect how some groups engage with the trial**

| **Eligibility** | How might eligibility criteria exclude members of each ethnic group in the target population for reasons other than their clinical eligibility for the trial (e.g. availability of medical history, must speak English, location, gender, age, discussing pregnancy, internet/mobile telephone access)? | Response: |  |
| --- | --- | --- | --- |
|  | Other factors to consider: | |  |
| **Opportunity to participate** | How might the way(s) (and by whom) potential participants are made aware of the trial (e.g. posters in clinic, written letter from a doctor, asked by a nurse) limit the participation of each ethnic group in the target population? | Response: |  |
|  | How might the information that tells potential participants about the trial (e.g. participant information leaflet) limit the participation of each ethnic group? | Response: |  |
|  | How might cultural practices, beliefs and traditions change the way each ethnic group perceives the information they are given? | Response: |  |
|  | Other factors to consider: | |  |
| **Consent procedures** | How might the way consent is sought (i.e. where, by whom, written vs verbal, verbal translations/multiple languages, access to interpreters) limit the participation of each ethnic group in the target population? | Response: | |
|  | How might the way people would like to discuss participation with family before providing consent differ for each ethnic group? | Response: | |
|  | How might the way the research team can check how well consent information is understood differ for each ethnic group? | Response: | |
|  | Other factors to consider: | | |

**Worksheet 3b**

This worksheet provides some questions **to guide your thinking about ethnic group involvement when answering Question 4** of the INCLUDE Key Questions.

**Trial data collection factors that might affect how some groups engage with the trial**

| **What** | How, and in what way, were people from each ethnic group in the target population involved in selecting the trial outcomes? | Response: |
| --- | --- | --- |
|  | How might the trial outcomes themselves, or other data being collected (e.g. a patient’s background information) limit the participation of each ethnic group? | Response: |
|  | Other factors to consider: | |
| **Who** | How might the people who collect data limit the participation of each ethnic group in the target population? | Response: |
|  | Other factors to consider: | |
| **How** | How might data collection methods limit the participation of each ethnic group in the target population? | Response: |
|  | Other factors to consider: | |
| **Where** | How might where data are collected limit the participation of each ethnic group in the target population? | Response: |
|  | Other factors to consider: | |

**Worksheet 3c**

This worksheet provides some questions **to guide your thinking about ethnic group involvement when answering Question 4** of the INCLUDE Key Questions.

**Factors that might affect the planned analysis of trial results**

| **Retention** | How might the trial data available for participants differ between each ethnic group in the target population? | Response: | |
| --- | --- | --- | --- |
|  | Other factors to consider: | | |
| **Benefits** | How might the benefits of the trial intervention(s) differ between each ethnic group in the target population? | Response: | |
|  | Other factors to consider: | | |
| **Harms** | How might the possible harms of the trial intervention(s) differ between each ethnic group in the target population? | Response: | |
|  | Other factors to consider: | | |
| **Subgroup analyses** | How should variation between ethnic groups in the target population be explored– should there be planned subgroup analyses? | Response: | |
|  | Other factors to consider: | | |
| **Interim analyses** | How should any interim analysis handle variation between ethnic groups in the target population? | | Response: |
|  | Other factors to consider: | | |
| **Stopping triggers** | How should any rules to stop the trial early on safety or benefit grounds handle variation between ethnic groups in the target population? | | Response: |
|  | Other factors to consider: | | |

**Worksheet 3d**

This this worksheet provides some questions **to guide your thinking about ethnic group involvement when answering Question 4** of the INCLUDE Key Questions.

**Factors that might affect the planned reporting and dissemination of trial results**

| **What** | How, and in what way, were people from each ethnic group in the target population involved in planning the reporting and dissemination of the trial results? | Response: |
| --- | --- | --- |
|  | Other factors to consider: | |
| **How** | How might planned reporting and dissemination methods limit engagement with each ethnic group in the target population? | Response: |
|  | Other factors to consider: | |
| **Where** | How might where trial results are planned to be reported and disseminated limit engagement of each ethnic group in the target population? | Response: |
|  | Other factors to consider: | |

Worksheet for thinking through measures to address factors that might prevent full community involvement

Use this worksheet to list key factors that might affect the involvement of some ethnic groups in the target population of your trial, along with measures to mitigate the effect of those factors and their cost. Add extra rows as needed.

Please remember that there are also differences *within* ethnic groups, especially between generations and between men and women. No ethnic group is homogenous.

| **Factors that may prevent full community involvement** | **Proposed measures (several options may be needed)*** | **Cost of measures** |
| --- | --- | --- |
|  |  |  |
|  |  |  |
|  |  |  |
|  |  |  |
|  |  |  |
|  |  |  |
|  |  |  |

*See https://centreforbmehealth.org.uk/resources/toolkits/ for suggestions for how to address factors that affect community-wide involvement.

Acknowledgements

In addition to [Trial Forge](https://www.trialforge.org/) and [NIHR](https://www.nihr.ac.uk/), this work has involved and been supported by the following:


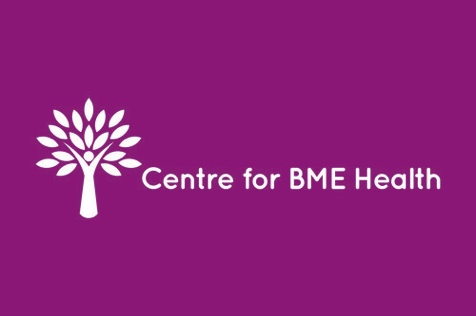


[Centre for Black and Minority Ethnic Health](https://centreforbmehealth.org.uk/)

**
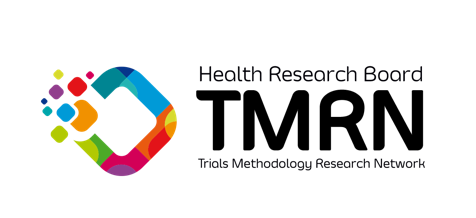
**

[Health Research Board Trial Methodology Research](https://www.hrb-tmrn.ie/)

[Network](https://www.hrb-tmrn.ie/)

**
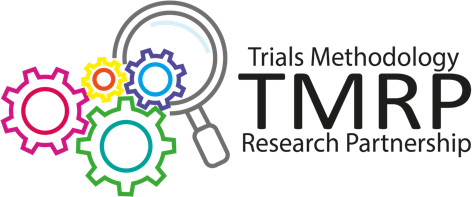
**[NIHR-Medical Research Council Trial Methodology](https://www.methodologyhubs.mrc.ac.uk/about/tmrp/)

[Research Partnership](https://www.methodologyhubs.mrc.ac.uk/about/tmrp/)

**
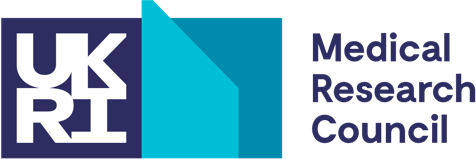
**[UK Research and Innovation & Medical Research](https://www.ukri.org/)

[Council](https://www.ukri.org/)


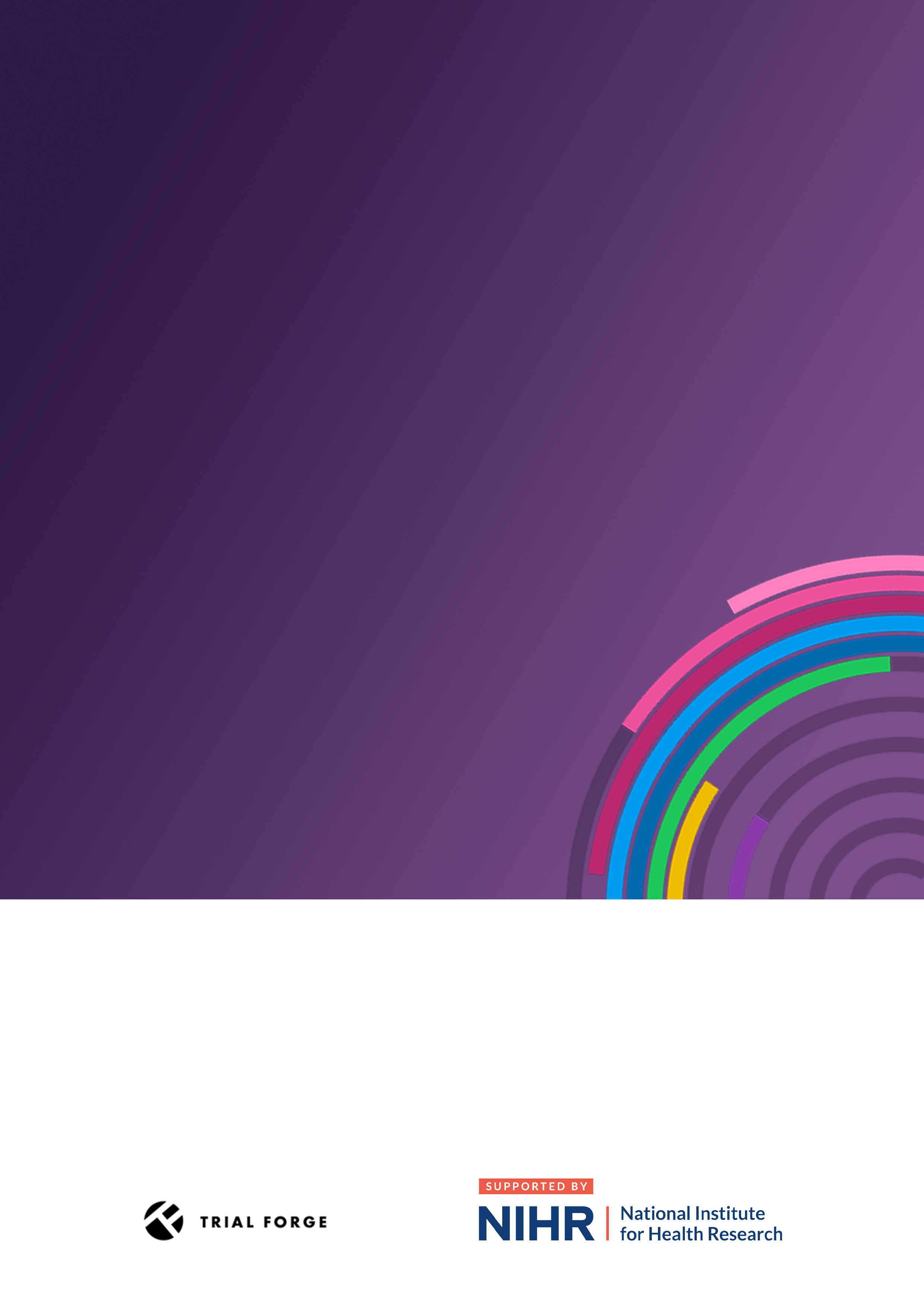

Supplement: Supplementary file 1 — Additional file 1. [file 13063_2021_5276_MOESM1_ESM.docx]
