# Supplementary material for: Developing the INCLUDE Ethnicity Framework—a tool to help trialists design trials that better reflect the communities they serve
Source: Trials. 2021 May 10;22:337. doi: 10.1186/s13063-021-05276-8 (PMC8108025; doi:10.1186/s13063-021-05276-8)
Supplement: Supplementary file 2 — Additional file 2. [file 13063_2021_5276_MOESM2_ESM.pdf]

How (and by who) was the INCLUDE Ethnicity Framework developed?

The National Institute for Health Research (NIHR) initiated the INCLUDE initiative in 2017. The Medical Research Council (MRC) Hubs for Trials Methodology Research Recruitment and Retention Working Group was at the same time starting efforts to improve representation within trials, particularly of black, Asian and minority ethnic individuals. The two groups came together in late 2018 to develop a research grant proposal for work on inclusion in trials. That grant originally included what has become the INCLUDE Ethnicity Framework, but the groups decided to develop the tool outside the grant in early 2019. Work on the INCLUDE Ethnicity Framework began in earnest in July 2019.

There were seven key phases between July 2019 and Sept 2020:

1. Developing an outline of what was needed
2. Developing an initial draft of the Framework
3. Discussing that draft with a wider stakeholder group
4. Modifying the draft in light of feedback from stakeholders.
5. Stakeholder feedback on the modified draft
6. Applying the Framework to 3-5 trials
7. Packaging the Framework, examples and other materials

In the summaries below we have listed the individuals who contributed to each phase, along with the perspective they brought described in their own words.

1. Developing an outline of what was needed (July 2019 - Oct 2019)

**Purpose**: to develop points for discussion at a meeting to be held in autumn 2019.		
**Participants**		
**Name**	**Affiliation**	**Perspective**
Peter Bower	MRC North West Hub for Trials Methodology Research, University of Manchester	Male, White British, Academic
Declan Devane	Health Research Board-Trials Methodology Research Network,<br>National University of Ireland Galway	Male, White Irish, Academic
Heidi Gardner	Health Services Research Unit, University of Aberdeen	White British woman, Researcher & Creative
Talia Isaacs	Centre for Applied Linguistics, University College London	Female, Mixed Other, Academic
Gary Nestor	NIHR Clinical Research Network Cluster E, University of Newcastle	Male, White British, Scientific Professional
Adwoa Parker	York Trials Unit, University of York	Female, Black British-Ghanaian, Academic
Lynn Rochester	NIHR Clinical Research Network Cluster E, University of Newcastle	Female, white British, clinical academic
Irene Soulsby	Public and patient representative, Newcastle	White British, Woman, Patient and Public Partner
Shaun Treweek	Health Services Research Unit, University of Aberdeen	Male, White British, Academic
Miles Witham	NIHR Clinical Research Network Cluster E, University of Newcastle	Male, White English, Academic, Clinician

2. Developing an initial draft of the Framework (Oct 2019 - Feb 2020)

Purpose: to discuss the outline from Phase 1 and then use this discussion to develop a full first draft of the INCLUDE Ethnicity Framework. Face-to-face meeting held in Newcastle on 2nd Oct 2019, attended by all below. The full draft coming from the meeting would be used in Phase 3.

Participants

**Name**	**Affiliation**	**Perspective**
Peter Bower	MRC North West Hub for Trials Methodology Research, University of Manchester	Male, White British, Academic
Declan Devane	Health Research Board-Trials Methodology Research Network,<br>National University of Ireland Galway	Male, White Irish, Academic
Heidi Gardner	Health Services Research Unit, University of Aberdeen	White British woman, Researcher & Creative
Talia Isaacs	Centre for Applied Linguistics, University College London	Female, Mixed Other, Academic
Gary Nestor	NIHR Clinical Research Network Cluster E, University of Newcastle	Male, White British, Scientific Professional
Adwoa Parker	York Trials Unit, University of York	Female, Black British-Ghanaian, Academic
Lynn Rochester	NIHR Clinical Research Network Cluster E, University of Newcastle	Female, white British, clinical academic
Irene Soulsby	Public and patient representative, Newcastle	White British, Woman, Patient and Public Partner
Shaun Treweek	Health Services Research Unit, University of Aberdeen	Male, White British, Academic
Miles Witham	NIHR Clinical Research Network Cluster E, University of Newcastle	Male, White English, Academic, Clinician

3. Discussing that draft with a wider stakeholder group (Feb 2020 - Mar 2020)

Purpose: to discuss the full draft of the Framework with a wider stakeholder group. A face-to-face meeting was held in London on 4th Feb 2020. All those present at the meeting are marked * in the table below. Email discussion post-meeting included everyone below. The summary of the meeting discussion was approved on 3rd March 2020. Work was then delayed by the outbreak of COVID-19.

Participants

Name	Affiliation	Perspective
Catherine Arundel*	York Trials Unit, University of York	White British female, Researcher
Peter Bower*	MRC North West Hub for Trials Methodology Research, University of Manchester	Male, White British, Academic
Helen Brittain	Research Ethic Committee– West Midlands – Coventry and Warwickshire	White - Jewish - female - Clinical psychologist & Chair of a REC
Declan Devane*	Health Research Board-Trials Methodology Research Network,<br>National University of Ireland Galway	Male, White Irish, Academic
Azhar Farooqi*	Centre for Black and Minority Ethnic Health, University of Leicester	British south Asian
Heidi Gardner*	Health Services Research Unit, University of Aberdeen	White British woman, Researcher & Creative
Talia Isaacs*	Centre for Applied Linguistics, University College London	Female, Mixed Other, Academic
Manos Kumar*	Public and patient representative, London	Concerned lay person from Colindale, North West London
Maisie McKenzie*	Public and patient representative, London	Black British Carer and Patient
Gary Nestor*	NIHR Clinical Research Network Cluster E, University of Newcastle	Male, White British, Scientific Professional
Adepeju Oshisanya *	Public and patient representative, London	Black African, British, Female, Researcher

Lynn Rochester	NIHR Clinical Research Network Cluster E, University of Newcastle	Female, white British, clinical academic
Laura Rooney*	Cancer Research UK, Beatson West of Scotland Cancer Centre	White Scottish female, Research Nurse
Irene Soulsby*	Public and patient representative, Newcastle	White British, Woman, Patient and Public Partner
Zareen Thorlu-Bangura*	Public and patient representative, London	Black African-British Female Student
Shaun Treweek*	Health Services Research Unit, University of Aberdeen	Male, White British, Academic
Unamed*	Public and patient representative	Preferred not to be named
Caroline Whiting*	James Lind Alliance, University of Southampton	White British woman, research management
Hywel Williams*	Centre of Evidence-Based Dermatology, University of Nottingham	White Welshman, research funder, anti-racist
Miles Witham	NIHR Clinical Research Network Cluster E, University of Newcastle	Male, White English, Academic, Clinician

4. Modifying the draft in light of feedback from stakeholders (June 2020 - July 2020)

Purpose: to modify the Feb 2020 draft to take account of comments at and post the 4th Feb London meeting. This work was re-started with funding from a UKRI-NIHR COVID-19 Rapid Response Initiative grant linked to ethnicity and COVID-19 trials; grantholders are marked with a * below.

Participants

**Name**	**Affiliation**	**Perspective**
Katie Banister	Health Services Research Unit, University of Aberdeen	White British, Academic
Seonaidh Cotton	Health Services Research Unit, University of Aberdeen	White Scottish woman, researcher
Heidi Gardner	Health Services Research Unit, University of Aberdeen	White British woman, Researcher & Creative
Gary Nestor*	NIHR Clinical Research Network Cluster E, University of Newcastle	Male, White British, Scientific Professional
Adepeju Oshisanya *	Public and patient representative, London	Black African, British, Female, Researcher
Lynn Rochester*	NIHR Clinical Research Network Cluster E, University of Newcastle	Female, white British, clinical academic
Irene Soulsby*	Public and patient representative, Newcastle	White British, Woman, Patient and Public Partner
Shaun Treweek*	Health Services Research Unit, University of Aberdeen	Male, White British, Academic
Miles Witham*	NIHR Clinical Research Network Cluster E, University of Newcastle	Male, White English, Academic, Clinician

5. Stakeholder feedback on the modified draft (July 2020 - Aug 2020)

**Purpose**: to invite feedback from stakeholders on the Phase 4 new draft, including all those who attended the 4th Feb meeting.		
**Participants**		
**Name**	**Affiliation**	**Perspective**
Magaly Aceves Martins	Health Services Research Unit, University of Aberdeen	Other ethnic group (Latin-American) woman, Researcher & Member of the public
Ella Anderson	NIHR Clinical Research Network Cluster E, University of Newcastle	White British woman, INCLUDE Project Coordinator
Catherine Arundel	York Trials Unit, University of York	White British female, Researcher
Alison Avenell	Health Services Research Unit, University of Aberdeen	White British woman, academic and NHS hospital consultant
Katie Banister	Health Services Research Unit, University of Aberdeen	White British, Academic
Peter Bower	MRC North West Hub for Trials Methodology Research, University of Manchester	Male, White British, Academic
Seonaidh Cotton	Health Services Research Unit, University of Aberdeen	White Scottish woman, researcher
Helen Brittain	Research Ethic Committee– West Midlands – Coventry and Warwickshire	White - Jewish - female - Clinical psychologist & Chair of a REC
Declan Devane	Health Research Board-Trials Methodology Research Network,<br>National University of Ireland Galway	Male, White Irish, Academic

Vikki Entwistle	Health Services Research Unit, University of Aberdeen	Academic, at home in Scotland, people would identify me as White (and awkwardly resistant to dividing people into tidy categories)
Heidi Gardner	Health Services Research Unit, University of Aberdeen	White British woman, Researcher & Creative
Katie Gillies	Health Services Research Unit, University of Aberdeen	White British woman, Researcher
Beatriz Goulao	Health Services Research Unit, University of Aberdeen	White Portuguese, Researcher
Jemma Hudson	Health Services Research Unit, University of Aberdeen	White British, Academic
Talia Isaacs	Centre for Applied Linguistics, University College London	Female, Mixed Other, Academic
Manos Kumar	Public and patient representative, London	Concerned lay person from Colindale, North West London
Kamlesh Khunti	Centre for Black and Minority Ethnic Health, University of Leicester	Male, Indian British, Academic
Louise Locock	Health Services Research Unit, University of Aberdeen	White British, researcher
Maisie McKenzie	Public and patient representative, London	Black British Carer and Patient
Gary Nestor	NIHR Clinical Research Network Cluster E, University of Newcastle	Male, White British, Scientific Professional
Avril Nicoll	Health Services Research Unit, University of Aberdeen	White Scottish qualitative researcher
Adepeju Oshisanya	Public and patient representative, London	Black African, British, Female, Researcher
Sahdia Parveen	Centre for Applied Dementia Studies, University of Bradford	Female, British Pakistani, Academic

Ash Rishi	Couch Health, Manchester	British born Indian, Clinical Trial Patient Recruitment and Retention
Lynn Rochester	NIHR Clinical Research Network Cluster E, University of Newcastle	Female, white British, clinical academic
Irene Soulsby	Public and patient representative, Newcastle	White British, Woman, Patient and Public Partner
Zareen Thorlu-Bangura	Public and patient representative, London	Black African-British Female Student
Shaun Treweek	Health Services Research Unit, University of Aberdeen	Male, White British, Academic
Unnamed	Public and patient representative	Preferred not to be named
Hywel Williams	Centre of Evidence-Based Dermatology, University of Nottingham	White Welshman, research funder, anti-racist
Miles Witham	NIHR Clinical Research Network Cluster E, University of Newcastle	Male, White English, Academic, Clinician

6. Applying the Framework to 3-5 trials (July 2020 - Aug 2020)

**Purpose**: to take the INCLUDE Ethnicity Framework and apply it to 3-5 trials. This was intended to give experience of using the Framework, as well as provide a small example set. This work was funded by the UKRI COVID-19 funding so three of the trials were COVID-19 trials.		
**Participants**		
**Name**	**Affiliation**	**Perspective**
Katie Banister	Health Services Research Unit, University of Aberdeen	White British, Academic
Seonaidh Cotton	Health Services Research Unit, University of Aberdeen	White Scottish woman, researcher
Heidi Gardner	Health Services Research Unit, University of Aberdeen	White British woman, Researcher & Creative
Maisie McKenzie	Public and patient representative, London	Black British Carer and Patient
Adepeju Oshisanya	Public and patient representative, London	Black African, British, Female, Researcher
Sahdia Parveen	Centre for Applied Dementia Studies, University of Bradford	Female, British Pakistani, Academic
Ash Rishi	Couch Health, Manchester	British born Indian, Clinical Trial Patient Recruitment and Retention
Irene Soulsby	Public and patient representative, Newcastle	White British, Woman, Patient and Public Partner
Shaun Treweek	Health Services Research Unit, University of Aberdeen	Male, White British, Academic
Miles Witham	NIHR Clinical Research Network Cluster E, University of Newcastle	Male, White English, Academic, Clinician

7. Packaging the Framework, examples and other materials (Aug 2020 - Sept 2020)

Purpose: to package the INCLUDE Ethnicity Framework, our guidance, examples, videos and other material so that it is easily accessible to trialists and others who may benefit from using it.

Participants

Name	Affiliation	Perspective
Ella Anderson	NIHR Clinical Research Network Cluster E, University of Newcastle	White British woman, INCLUDE Project Coordinator
Katie Banister	Health Services Research Unit, University of Aberdeen	White British, Academic
Michael Bonar	Centre for Black and Minority Ethnic Health, University of Leicester	Male, White British, Creative
Seonaidh Cotton	Health Services Research Unit, University of Aberdeen	White Scottish woman, researcher
Kim Down	NIHR Clinical Research Network Cluster E, University of Newcastle	White Canadian, INCLUDE Project Manager
Charlie Franklin	Centre for Black and Minority Ethnic Health, University of Leicester	Male, White British, Creative
Heidi Gardner	Health Services Research Unit, University of Aberdeen	White British woman, Researcher & Creative
Talia Isaacs	Centre for Applied Linguistics, University College London	Female, Mixed Other, Academic
Gary Nestor	NIHR Clinical Research Network Cluster E, University of Newcastle	Male, White British, Scientific Professional
Robert O'Halloran	Rob&Paul Digital Design	White Irish, Developer
Paul Gallagher	Rob&Paul Digital Design	White British, Designer
Adepeju Oshisanya	Public and patient representative, London	Black African, British, Female, Researcher
Lynn Rochester	NIHR Clinical Research Network Cluster E, University of Newcastle	Female, white British, clinical academic

Irene Soulsby	Public and patient representative, Newcastle	White British, Woman, Patient and Public Partner
Shaun Treweek	Health Services Research Unit, University of Aberdeen	Male, White British, Academic
Miles Witham	NIHR Clinical Research Network Cluster E, University of Newcastle	Male, White English, Academic, Clinician
