# Supplementary material for: Developing the INCLUDE Ethnicity Framework—a tool to help trialists design trials that better reflect the communities they serve
Source: Trials. 2021 May 10;22:337. doi: 10.1186/s13063-021-05276-8 (PMC8108025; doi:10.1186/s13063-021-05276-8)
Supplement: Supplementary file 3 — Additional file 3. [file 13063_2021_5276_MOESM3_ESM.pdf]

Ethnic group categorisation/criteria – some examples for discussion

Race and ethnicity

It is important to attempt to outline the concepts of 'race' and 'ethnicity', since both terms are used by government institutions to categorise their populations.

As a group, we need to consider how important it is to make a clear distinction between race and ethnicity

Defining ethnicity

Ethnicity has been defined as: 'the social group a person belongs to, and either identifies with or is identified with by others, as a result of a mix of cultural and other factors including language, diet, religion, ancestry and physical features traditionally associated with race' (Bhopal, 2004, p. 442).

The characteristics that define ethnicity are not fixed or easily measured, so ethnicity is imprecise and fluid. Ethnicity differs from race, nationality, religion, and migrant status, sometimes in subtle ways, but may include facets of these other concepts. Researchers who wish to study ethnicity should collect data on such underlying factors, especially language, religion, country of birth, and family origins (Bhopal 2004).

Defining race

The concept of 'race' is controversial. It is difficult to define a rationale for racial categories and there is no consistent agreement about an objective set of categories.

'Race provides a way of defining, for social purposes, populations that look different and have different ancestral roots' (Bhopal 2004).

From (Bhopal 2004): The biological concept of race, whereby human populations were divided into sub-species mainly on the basis of visible physical characteristics, was dominant from the early 19th century to its decline with the defeat of the Nazis at the end of World War II (Senior, 1994). None of the numerous racial classifications have stood the test of time, though there are echoes in current classifications. In retrospect, the biological concept of race was ill defined, poorly understood, and invalid and the science based on it needed sharper scientific criticism (Bhopal 1994). The modern concept of race, emphasises its social origins rather than its biological basis (Kaplan 2003). In this perspective, race provides a way of defining, for social purposes, populations that look different and have different ancestral roots. It is evident, however, than even this social concept of race is ultimately based on physical and hence biological factors, though these are de-emphasised in social epidemiology as secondary, unimportant matters. The term race should be used with caution for its history is one of misuse and injustice.

A note on categories and classifications of race

From (Bhopal 2004): To put the concepts of race and ethnicity into operation we need to derive categories, as most clearly seen in national censuses. Researchers have mostly used such administrative categories for race and ethnicity, even when these are acknowledged by those developing them as having no scientific or anthropological validity. Scientists' use of existing

classifications can be interpreted as an endorsement of their validity. As a minimum, researchers should explain their understanding of the concepts of race or ethnicity and the classification they use (for example, one or a mix of ancestry, geographical origin, birthplace, language, religion, migration history, name, self-identity, observation, etc).

Methods for identifying these examples.

I initially reviewed the ethnicity categories used in the latest household census conducted in England and Wales by the Office for National Statistics (2011: https://www.ons.gov.uk/census/2011census). I then reviewed the categories used by UK state institutions; namely the NHS and the criminal justice system. I then specifically looked at how ethnicity data are captured in census data in Scotland, Wales and Northern Ireland, to determine if there were differences in ethnicity data capture.

Following this, I looked at the Republic of Ireland, and then other English speaking OECD countries with high migrant populations. I started with OECD countries since these are relatively similar to the UK and Ireland. The following countries were reviewed:

- Australia
- Canada
- New Zealand
- United States

Other OECD countries with high immigrant populations such as France, Germany, Sweden and The Netherlands do not collect statistics on ethnicity or race. Rather, proxy terms such as *nationality, language most often spoken at home, migration background (country of birth and citizenship of individuals and of parents), origin (e.g. foreign origin vs Swedish Origin), and religion and belief* are used to gather data on their populations (Farkas, 2017). These countries have therefore not been included here.

UK Harmonised Concepts and Questions for Social Data Sources: Ethnic Group

In the UK, there are Harmonised Concepts and Questions for Social Data Sources for gathering ethnic group data, which has been developed by the Office for National Statistics (ONS, 2015). Harmonised ethnic group questions have been used since 2001. This sets out the harmonised country specific ethnic group questions for use in social surveys in England, Wales, Scotland and Northern Ireland. Ethnic group is classified according to the person's own perceived ethnic group and cultural background. This covers face-to-face surveys, self-completed surveys and telephone surveys. They have been developed through consultation and workshops with key stakeholders that have included:

- Scottish Government (SG) / National Records of Scotland (NRS)
- Welsh Government (WG)
- Office of the First Minister & Deputy First Minister/ Northern Ireland Statistics and Research Agency
- Equality and Human Rights Commission
- Data Standards Working Group
- Other government departments
- Academic

Some ethnicity classifications vary between countries due to the specific legal requirements:

- For **Scotland**, specific requirements came out of a review of the way Scottish surveys classified ethnicity following recommendations made by the Race Equality Advisory Forum in 2001 and community concerns about the classification used in Scotland's 2001 Census. The review was conducted by SG and NRS and identified the benefits for developing a separate national identity and new ethnicity classification for use on the Scottish Census and relevant Scottish Official Statistics.
  Both questions used together would allow people to self-express their 'Scottish-ness', 'British-ness' or any other national identity before expressing their ethnic origin. The new ethnicity classification was published in July 2008 and details of the classification can be found at http://www.scotland.gov.uk/Publications/2008/07/29095058/0. The classification was discussed by Members of the Scottish Parliament during considerations of the 2011 Census in Scotland and was subsequently amended for use in the Census.
- For **Northern Ireland** specific requirements comply with the Good Friday Agreement (where it isn't acceptable to ask respondents to choose between 'Northern Irish/British' and 'Irish identities') and legislation under the Race Relations (NI) Order 1997 which outlaws discrimination on grounds of colour, race, nationality or ethnic or national origin. The Irish Traveller community is specifically identified in the Order as a racial group against which racial discrimination is unlawful.

The harmonised ethnicity question is used for the UK census, and also forms the basis of the NHS ethnicity questions, and the ethnicity questions used in the criminal justice system.

Harmonised country specific ethnic group question: England

Ethnic group classifies people according to their own perceived ethnic group and cultural background. Below is the recommended ethnic group question for use in England. Note this is the same wording used in the 2011 Census:

This question is recommended when a show card is used in a face to face interview or self completion survey (both paper and electronic).

'What is your ethnic group?

Choose one option that best describes your ethnic group or background'

**Ethnic Group**
White
British (English/Welsh/Scottish/Northern Irish/British)
Irish
Gypsy or Irish Traveller
Any other White background, *please describe*
Mixed/Multiple ethnic group
White and Black Caribbean
White and Black African
White and Asian
Any other Mixed / Multiple ethnic background, *please describe*
Asian/Asian British
Indian
Pakistani

Bangladeshi
Chinese
Any other Asian background, *please describe*
Black/African/Caribbean/Black British
African
Caribbean
Any other Black / African / Caribbean background, *please describe*
Other ethnic group
Arab
Any other ethnic group, *please describe*

Harmonised country specific ethnic group question: Wales

Below is the recommended ethnic group question for use in Wales. This question is recommended when a show card is used in a face to face interview or self completion survey (both paper and electronic).

This question is recommended when a show card is used in a face to face interview or self completion survey (both paper and electronic).

'What is your ethnic group?

Choose one option that best describes your ethnic group or background'

**Ethnic Group**
White
Welsh / English / Scottish / Northern Irish / British
Irish
Gypsy or Irish Traveller
Any other White background, please describe
Mixed/Multiple ethnic group
White and Black Caribbean
White and Black African
White and Asian
Any other Mixed / Multiple ethnic background, *please describe*
Asian/Asian British
Indian
Pakistani
Bangladeshi
Chinese
Any other Asian background, *please describe*
Black/African/Caribbean/Black British
African
Caribbean
Any other Black / African / Caribbean background, *please describe*

Other ethnic group
Arab
Any other ethnic group, please describe

Harmonised country specific ethnic group question: Scotland

Below is the recommended ethnic group question for use in Scotland. This question has been developed to enable direct comparison with the Scottish Census and other sources in Scotland.

This question is recommended when a show card is used in a face to face interview or self completion survey (both paper and electronic).

'What is your ethnic group?

Choose one option that best describes your ethnic group or background'

**Ethnic Group**
White
Scottish
Other British
Irish
Gypsy or Irish Traveller
Polish
Any other White ethnic group, *please describe*
Mixed/Multiple ethnic group
Any Mixed or Multiple ethnic groups, *please describe*
Asian/Asian British
Pakistani, Pakistani Scottish or Pakistani British
Bangladeshi, Bangladeshi Scottish or Bangladeshi British
Chinese, Chinese Scottish or Chinese British
Any other Asian, *please describe*
African
African, African Scottish or African British
Any other African, *please describe*
Caribbean or Black
Caribbean, Caribbean Scottish or Caribbean British
Black, Black Scottish or Black British
Any other Caribbean or Black, *please describe*
Other ethnic group
Arab, Arab Scottish or Arab British
Any other ethnic group, please describe

Harmonised country specific ethnic group question: Northern Ireland

Below is the recommended ethnic group question for Northern Ireland.

This question is recommended when a show card is used in a face to face interview or self completion survey (both paper and electronic).

'What is your ethnic group?

Choose one option that best describes your ethnic group or background'

Ethnic Group
White
Irish Traveller
Any other White background, *please describe*
Mixed/Multiple ethnic group
White and Black Caribbean
White and Black African
White and Asian
Any other Mixed / Multiple ethnic background, *please describe*
Asian/Asian British
Indian
Pakistani
Bangladeshi
Chinese
Any other Asian background, *please describe*
Black/African/Caribbean/Black British
African
Caribbean
Any other Black / African / Caribbean background, *please describe*
Other ethnic group
Arab
Any other ethnic group, *please describe*

NHS Ethnic category codes

The NHS ethnic category codes are based on the harmonised ethnic group question, specifically that used in the 2001 ONS census. This as for the ethnicity of a PERSON, as specified by the PERSON. (Source:www.datadictionary.nhs.uk/data_dictionary/attributes/e/end/ethnic_category_code_de.asp)

National Codes:	
White	
A	British
B	Irish
C	Any other White background
Mixed	
D	White and Black Caribbean
E	White and Black African
F	White and Asian
G	Any other mixed background
Asian or Asian British	
H	Indian
J	Pakistani
K	Bangladeshi
L	Any other Asian background
Black or Black British	
M	Caribbean
N	African
P	Any other Black background
Other Ethnic Groups	
R	Chinese
S	Any other ethnic group
Z	Not stated

'National code Z - Not Stated should be used where the PERSON has been given the opportunity to state their ETHNIC CATEGORY but chose not to'.

HM Government Criminal Justice System: Introduction of 'Self Defined Ethnicity – 18+1' Standard

The HM Government Criminal Justice System recently introduced 'self defined ethnicity' (HM Government 2018). The NHS ethnic category codes are based on the harmonised ethnic group question, specifically based on the ONS 16+1 ETHNICITY code as used in the 2011 census.

W1 British
W2 Irish
W3 Gypsy or Irish Traveller
W9 Any other white background
M1 White and Black Caribbean
M2 White and Black African
M3 White and Asian
M9 Any other mixed
A1 Indian
A2 Pakistani
A3 Bangladeshi
A4 Chinese
A9 Any other Asian background
B1 Caribbean
B2 African
B9 Any other Black background
O2 Arab
O9 Any other
NS Not stated

Republic of Ireland

A question on ethnic or cultural background was asked for the first time in the Irish census in 2006 (Central Statistics Office 2016). According to King-O'Riain (2007), the capture of ethnicity data in the Irish census was influenced by international censuses (particularly from the UK with which it shares a common travel area), the historical ethnicisation of Travellers (there has been a long-standing debate about whether Travellers, a disadvantaged indigenous nomadic group, are considered `ethnic' or not) and increasing awareness of ethnocultural characteristics among European statistical agencies.

The 2016 Census asks respondents to self-f identify their ethnicity. (https://www.cso.ie/en/media/csoie/census/census2016/2016censusforms/65995_English_Household_2016_New_Version_Do_Not_Complete.pdf)

What is your ethnic or cultural background?

Choose ONE section from A to D, then the appropriate box

**A White**
White Irish
White Irish Traveller
Any other white background
**B Black or Black Irish**
Black or Black Irish - African
Black or Black Irish - Any other Black Background
**C Asian or Asian Irish**
Asian or Asian Irish - Chinese
Asian or Asian Irish - Any other Asian Background
**D Other (including mixed background)**
Other, write in description.

Australia

Australian Bureau of Statistics (ABS) Australian Standard Classification of Cultural and Ethnic Groups is the Australian statistical standard for classifying statistics by cultural and ethnic groups (ASCCEG 2016). ASCCEG is designed to be used for the classification of information relating to topics such as ancestry, ethnic identity, and cultural diversity.

Definition of ethnicity

ASCCEG is designed to be used for the classification of information relating to topics such as ancestry, ethnic identity, and cultural diversity. Although these topics have elements of difference, it is considered that the concept common to them all, and underpinning the classification, is ethnicity.

The words 'ethnicity' and 'ethnic' are associated with many different meanings. The *Macquarie Dictionary* (on line Edition 2016) provides the following Australian context:
1. relating to or peculiar to a human population or group, especially one with a common ancestry, language, etc
2. relating to the origin, classification, characteristics, etc, of such groups
3. of or relating to members of the Australian community who are migrants or the descendants of migrants and whose first language is not English
4. recognisable as coming from an identifiable culture.

For the purposes of ASCCEG, 'ethnicity' refers to the shared identity or similarity of a group of people on the basis of one or more factors. These factors were enunciated by the 1986 Population Census Ethnicity Committee in *The Measurement of Ethnicity in the Australian Census of Population and Housing* report to the Australian Statistician (the Borrie Report). "The Committee considered that the most enlightening attempt to define an ethnic group is that contained in a United Kingdom Law Lords statement" (reported in Patterns of Prejudice, Vol 17, No. 2, 1983). The Law Lords noted the key factor as being the group regarding itself and is regarded by others, as a distinct community by virtue of certain characteristics, not all of which have to be present in the case of each ethnic group.

Several distinguishing characteristics were cited, including:

- a long shared history, the memory of which is kept alive
- a cultural tradition, including family and social customs, sometimes religiously based
- a common geographic origin
- a common language (but not necessarily limited to that group)
- a common literature (written or oral)
- a common religion
- being a minority (often with a sense of being oppressed)
- being racially conspicuous.

Since publication of the Borrie Report in 1984, the multicultural nature of Australian society has further developed; however, the approach to the definition of ethnicity in the Borrie Report is still relevant and serves the purposes of ASCCEG.

The approach of defining ethnic or cultural groups in terms of one or more relevant characteristics allows the notion of ethnicity to be viewed in broad sub-concepts. The Borrie Report describes these as a self perceived group identification approach, and an approach that is more historically determined. In ASCCEG, ethnicity is based on the self perceived group identification approach for a number of reasons:

- Self perceived group identification measures the extent to which individuals associate with particular cultural or ethnic groups. A measure of active association produces data which is more useful in terms of policy and service delivery needs. An historically determined approach would produce data which relates individuals to groups with which they no longer have a particular affinity and with which they may have little social, cultural or economic similarity.
- The method used to collect information on ethnicity or ancestry in the ABS and other organisations is self-perception based on a self assessed response to a direct question. No attempt is made to historically determine the origins of individuals. It is important for the concept underpinning the classification and the categories of the classification to be in harmony with this approach.
- The use of self-perception results in the need to include a number of categories in the classification that equate to national cultural identities e.g. Australian.

Considering ethnicity as a multi dimensional concept based on a number of distinguishing characteristics using a self-perception approach allows for a practical and useful classification attuned to generally accepted notions of what constitutes ethnicity and cultural identity. This approach supports the collection and use of data in statistical, administrative and service delivery settings.

Australian 2016 Census questions

11 Is the person an Australian citizen?

- Yes, Australian citizen
- No

12 In which country was the person born?

- Australia
- England
- New Zealand
- India
- Italy
- Vietnam
- Philippines
- Other (please specify)

13 In what year did the person first arrive in Australia to live here for one year or more? 18 What is the person's ancestry?

14 In which country was the person's father born?

- Australia
- Other (please specify)

15 In which country was the person's mother born?

- Australia
- Other (please specify)

18 What is the person's ancestry?

- Provide up to two ancestries only.
- Examples of 'Other': GREEK, VIETNAMESE, HMONG, KURDISH, MAORI, LEBANESE, AUSTRALIAN SOUTH SEA ISLANDER
    - English
    - Irish
    - Scottish
    - Italian
    - German
    - Chinese
    - Australian
    - Other ancestry 1 (please specify)
    - Other ancestry 2 (please specify)

United States

Unlike that used by other countries, the US Government's census focuses on race, not just ethnicity. Interestingly, Ethnicity in the US census is only classed as 'Hispanic or Latino' and 'Not Hispanic or Latino'. https://www.census.gov/mso/www/training/pdf/race-ethnicity-onepager.pdf

What is race?

The Census Bureau defines race as a person's self-identification with one or more social groups. An individual can report as White, Black or African American, Asian, American Indian and Alaska Native, Native Hawaiian and Other Pacific Islander, or some other race. Survey respondents may report multiple races.

What is ethnicity?

Ethnicity determines whether a person is of Hispanic origin or not. For this reason, ethnicity is broken out in two categories, Hispanic or Latino and Not Hispanic or Latino. Hispanics may report as any race.

What is Race?

'The data on race were derived from answers to the question on race that was asked of individuals in the United States. The Census Bureau collects racial data in accordance with guidelines provided by the U.S. Office of Management and Budget (OMB), and these data are based on self-identification.

The racial categories included in the census questionnaire generally reflect a social definition of race recognized in this country and not an attempt to define race biologically, anthropologically, or genetically. In addition, it is recognized that the categories of the race item include racial and national origin or sociocultural groups. People may choose to report more than one race to indicate their racial mixture, such as "American Indian" and "White." People who identify their origin as Hispanic, Latino, or Spanish may be of any race.

OMB requires five minimum categories: White, Black or African American, American Indian or Alaska Native, Asian, and Native Hawaiian or Other Pacific Islander'.

'An individual's response to the race question is based upon self-identification. The Census Bureau does not tell individuals which boxes to mark or what heritage to write in. For the first time in Census 2000, individuals were presented with the option to self-identify with more than one race and this continued with the 2010 Census. People who identify with more than one race may choose to provide multiple races in response to the race question'.
https://www.census.gov/topics/population/race/about.html

United States 2020 planned census questions

NOTE: please answer both question 8 about Hispanic origin and Question 9 about race. For this census, Hispanic Origins are not races.

8. Is Person 1 of Hispanic, Latino, or Spanish Origin?

- No, not Hispanic, Latino or Spanish Origin
- Yes, Mexican, Mexican Am., Chicano
- Yes, Puerto Rican
- Yes, Cuban
- Yes, Another Hispanic, Latin or Spanish Origin – *print, for example, Salvadoran. Dominican, Colombian, Guatemalan, Spaniard, Ecuadorian, etc.*

9. What is Person 1's race?

Mark X one or more boxes AND print Origins

- White – *print, for example, German, Irish, English, Italian, Lebanese, Egyptian, etc.*

- Black or African Am. – *print, for example, African American, Jamaican, Haitian, Nigerian, Ethiopian, Somali, etc.*
- American Indian or Alaskan Native – *print name of enrolled or principal tribes, for example Navajo Nation, Blackfeet Tribe, Mayan, Aztec, etc.*
- Chinese
- Filipino
- Asian Indian
- Vietnamese
- Korean
- Japanese
- Other Asian – *print, for example, Pakistani, Cambodian, etc.*
- Native Hawaiian
- Samoan
- Chamorro
- Other Pacific Islander – *print, for example, Tongan, Fijian, etc.*

Canada: 2016 Census of Population questions

The Canadian Census takes a slightly different approach, and may perhaps be more nuanced than some of the census categories used by other countries:

According to statistics Canada, who undertook the 2016 Canadian census, the 2016 Census of Population question on ethnic origin collects information on the ancestral origins of the population and provides information about the composition of Canada's diverse population.

Ethnic origin refers to the ethnic or cultural origins of the person's ancestors. An 'ancestor' is usually more distant than a grandparent. Other than Aboriginal persons, most people can trace their origins to their ancestors who first came to this continent. A person may have only a single ethnic origin, or may have multiple ethnicities.

Ethnic origin refers to a person's 'roots' and should not be confused with citizenship, nationality, language or place of birth. For example, a person who has Canadian citizenship, speaks Punjabi (Panjabi) and was born in the United States may report Guyanese ethnic origin.

It is important to note that ethnic origin responses are a reflection of each respondent's perception of their ethnic ancestry. Consequently, the measurement of ethnicity is affected by changes in the social environment in which the question is asked and changes in the respondent's understanding or views about the topic. Awareness of family background or length of time since immigration can affect responses to the ethnic origin question as well.

This means that two respondents with the same ethnic ancestry could have different response patterns and thus could be counted as having different ethnic origins. For example, a respondent could report 'East Indian' as an ethnic origin while another respondent, with a similar ancestral background, could report 'Punjabi' or 'South Asian' instead. Therefore, ethnic origin data can be fluid. Nevertheless, ethnic origin data in the Census of Population are a reflection of the respondent's perception of his or her ethnic ancestry at the time of collection. Users who wish to obtain broader response estimates may wish to combine data for one or more ethnic origins together or use estimates for ethnic categories (e.g., 'South Asian origins').

In the 2016 Census, the terms 'ethnic origin,' 'ethnic group' and 'ethnic ancestry' are used interchangeably

Since the 1981 Census, Canadians have been asked to report all the ethnic and cultural origins of their ancestors themselves, both on their paternal and maternal sides. Up to six origins per person were retained in 2016.

The 2016 Census includes data for more than 250 ethnic origins reported by people living in Canada. For each ethnic origin published, total, single and multiple response counts are provided.

A single ethnic origin response occurs when a respondent provides one ethnic origin only. For example, in 2016, about 475,575 people stated that their only ethnic origin was Scottish.

A multiple response occurs when a respondent provides two or more ethnic origins. For example, in 2016, about 4,323,430 people gave a response which included Scottish and one or more other ethnic origins (https://www12.statcan.gc.ca/census-recensement/2016/ref/guides/008/98-500-x2016008-eng.cfm).

This question collects information on the ancestral origins of the population and provides information about the composition of Canada's diverse population.

17. What were the ethnic or cultural origins of this person's ancestors?

    An ancestor is usually more distant than a grandparent.

    For example, Canadian, English, Chinese, French, East Indian, Italian, German, Scottish, Cree, Mi'kmaq, Salish, Métis, Inuit, Filipino, Irish, Dutch, Ukrainian, Polish, Portuguese, Vietnamese, Korean, Jamaican, Greek, Iranian, Lebanese, Mexican, Somali, Colombian, etc.

    - 1: Specify as many origins as applicable using capital letters.

18. STEP E - Question identifier 18:Is this person an Aboriginal person, that is, First Nations (North American Indian), Métis or Inuk (Inuit)?
    Note: First Nations (North American Indian) includes Status and Non-Status Indians.

    If "Yes", mark the circle(s) that best describe(s) this person now.

    - 1: No, not an Aboriginal person. Continue with the next question.
    - 2: Yes, First Nations (North American Indian). Go to question 20.
    - 3: Yes, Métis. Go to question 20.
    - 4: Yes, Inuk (Inuit). Go to question 20.

This question collects information in accordance with the *Employment Equity Act* and its Regulations and Guidelines to support programs that promote equal opportunity for everyone to share in the social, cultural, and economic life of Canada.

19. STEP E - Question identifier 19:Is this person:

    Mark more than one circle or specify, if applicable.

    - 1: White
    - 2: South Asian (e.g., East Indian, Pakistani, Sri Lankan, etc.)
    - 3: Chinese
    - 4: Black
    - 5: Filipino
    - 6: Latin American
    - 7: Arab
    - 8: Southeast Asian (e.g., Vietnamese, Cambodian, Laotian, Thai, etc.)

- 9: West Asian (e.g., Iranian, Afghan, etc.)
- 10: Korean
- 11: Japanese
- 12: Other — specify

20. STEP E - Question identifier 20:Is this person a Status Indian (Registered or Treaty Indian as defined by the *Indian Act* of Canada)?
    - 1: No
    - 2: Yes, Status Indian (Registered or Treaty)
21. STEP E - Question identifier 21:Is this person a member of a First Nation/Indian band? If " Yes ", which First Nation/Indian band?

    For example, Musqueam Indian Band, Sturgeon Lake First Nation, Atikamekw of Manawan.

    - 1: No
    - 2: Yes, member of a First Nation/Indian band. Specify name of First Nation/Indian Band.

Source: https://www12.statcan.gc.ca/nhs-enm/2016/ref/questionnaires/questions-eng.cfm

New Zealand

According to the Stats New Zealand, who undertook the 2013 census, ethnicity classification is based on the ethnic groups that people feel that they belong to (https://www.stats.govt.nz/topics/ethnicity).

Ethnicity definition: 'Ethnicity is a measure of cultural affiliation. It is not a measure of race, ancestry, nationality, or citizenship. Ethnicity is self perceived and people can belong to more than one ethnic group.

An ethnic group is made up of people who have some or all of the following:

- a shared culture, such as traditions, customs, beliefs, or language
- a common ancestry or history
- a similar geographic, tribal, or clan origin.

The census asks people to state the ethnic group or groups they belong to. People can select ethnic groups from a list and write in additional ethnicities. Each ethnic group includes all those who have identified with it, so people may be counted in more than one group. (Source: https://www.stats.govt.nz/tools/2013-census-ethnic-group-profiles)

In ethnic group (grouped total responses) a person belonging to more than one ethnic group is counted once in each group. For example, at level one of this classification, a person of Samoan, Tongan, and German ethnicity would be counted once in the category of Pacific peoples and once as European.

Which ethnic group do you belong to?

Mark the space or spaces which apply to you.

- New Zealand European
- Mäori
- Samoan
- Cook Island Maori

- Tongan
- Niuean
- Chinese
- Indian
- other such as DUTCH, JAPANESE, TOKELAUAN. Please state:

References

Arshad, R. (2010). Race Equality Toolkit for Learning and Teaching. Web publication/site, Universities Scotland. Retrieved from http://www.universities-scotland.ac.uk/raceequalitytoolkit/

Australian Standard Classification of Cultural and Ethnic Groups (ASCCEG) (2016). The Australian statistical standard for classifying statistics by cultural and ethnic groups: https://www.abs.gov.au/ausstats/abs@.nsf/mf/1249.0)

Bhopal RS. Is research into ethnicity and health racist, unsound, or important science? *BMJ*1997;**314**:1751–6

Bhopal, R. (2004). "Glossary of terms relating to ethnicity and race: for reflection and debate."

Burchard EG, Ziv E, Coyle N, Gomez SL, Tang H, Karter AJ, Mountain JL, Perez-Stable EJ, Sheppard D, Risch N. The importance of race and ethnic background in biomedical research and clinical practice. New England Journal of Medicine 2003;348:1170-5.

Central Statistics Office (2016). Census 2016 Summary Results - Part 1. Published by the Central Statistics Office, Ireland April 2017. https://www.cso.ie/en/media/csoie/newsevents/documents/census2016summaryresultspart1/Census2016SummaryPart1.pdf

Cooper RS, Kaufman JS, Ward R. Race and genomics. New England Journal of Medicine 2003;348(12):1166-70.

Farkas, L (2017). Analysis and comparative review of equality data collection practices in the European Union: Data collection in the field of ethnicity. EUROPEAN COMMISSION. Directorate-General for Justice and Consumers Directorate D — Equality Unit JUST/D1

Goodfriend, W. (2010). Race and Ethnicity Definitions: Social Minority vs. Social Majority, Lecture Material, Buena Vista University, in: “Intro to Sociology/Introducing Race & Diversity“, p. 26.

HM Government (2018). Criminal Justice System Exchange Data Standards Catalogue. Notification of Change: Introduction of ‘Self Defined Ethnicity – 18+1’ Standard. Version 1. Approval Date 01/03/2018

https://assets.publishing.service.gov.uk/government/uploads/system/uploads/attachment_data/file/691544/self-defined-ethnicity-18plus1.pdf

King-O’Riain, R. C. (2007). Counting on the `Celtic Tiger’: Adding ethnic census categories in the Republic of Ireland. Ethnicities, 7(4), 516–542. https://doi.org/10.1177/1468796807084015

Office for National Statistics (2015). Harmonised Concepts and Questions for Social Data Sources Primary Principles: Ethnic Group. Version 3.3 Office for National Statistics, May 2015 © Crown Copyright. file:///C:/Users/ahm521/Downloads/p3ethnicgroupmay15_tcm77-181143.pdf

Risch N, Burchard E, Ziv E, Tang H. Categorization of humans in biomedical research: genes, race and disease. Genome Biology 2002;3:2007.

Rosenberg NA, Pritchard JK, Weber JL, et al. Genetic structure of human populations. Science 2002;298:2381-5.

Senior P, Bhopal RS. Ethnicity as a variable in epidemiological research. *BMJ*1994;**309**:327–9.
