# Supplementary material for: Developing the INCLUDE Ethnicity Framework—a tool to help trialists design trials that better reflect the communities they serve
Source: Trials. 2021 May 10;22:337. doi: 10.1186/s13063-021-05276-8 (PMC8108025; doi:10.1186/s13063-021-05276-8)
Supplement: Supplementary file 4 — Additional file 4. [file 13063_2021_5276_MOESM4_ESM.pdf]

Trial features to consider

Key feature	Question	Notes
Prevalence of the disease or condition targeted	What is the prevalence of the targeted disease or condition in this ethnic group?<br>Is this different from the prevalence in the general population?<br>Is the severity of the disease different in this ethnic group?	
Awareness of the trial	Referrals and relationships:<br>Who is referring participants to the trial, and do potential participants have an existing relationship with this individual or team?	Link with continuity of care, trust, respect<br><br>Are these staff members able to provide culturally congruent and linguistically appropriate care?
Opportunity to participate	Eligibility:<br>Are there eligibility criteria that are likely to exclude members of this ethnic group for reasons other than their clinical eligibility for the trial?	'Must speak English', or 'must have an internet connection' may exclude some members of ethnic minority groups – reasons other than their clinical eligibility<br><br>Co-morbidities or health issues/experiences that are more common in certain minority groups
	Informed consent:<br>How is trial information being delivered to potential participants (mode of delivery – verbal/translations/multiple languages) and are there barriers that may prevent this ethic group from being given a true opportunity to participate?	What languages are trial materials (consent form, participant information leaflet, educational materials, research protocols) available in? Are there barriers to getting these editions or are they just as readily available as English editions?<br><br>Availability of bilingual staff<br><br>Cultural issues – direct eye contact, especially between two people of different age range, is considered rude (Asian culture), direct eye

		contact is a sign of honesty and attentiveness etc.
Acceptance of invitation to participate	Informed consent:<br>What information are you giving to potential participants, and is this information culturally relevant?	Information on gender segregation, cultural customs and religious sensitivity<br><br>Literacy levels<br><br>Can you engage the community in the development of research materials?<br><br>Family diversity that's different from yours – tension around decision-making – non-verbal cues
	Data collection:<br>How are data collected and could these methods impact on this ethnic group more than another?	Location, method (language translations for an app for example, literacy in terms of languages, but also IT literacy)
	The trial intervention	Cultural sensitivity to views around healthcare – e.g. a surgical scar stays with you into your next lives, and it is better to have a shorter life than to be scarred forever
