# Supplementary material for: Developing the INCLUDE Ethnicity Framework—a tool to help trialists design trials that better reflect the communities they serve
Source: Trials. 2021 May 10;22:337. doi: 10.1186/s13063-021-05276-8 (PMC8108025; doi:10.1186/s13063-021-05276-8)
Supplement: Supplementary file 5 — Additional file 5. [file 13063_2021_5276_MOESM5_ESM.pdf]

Trial participants: ethnicity (draft 17/1/2020)

1. Are there known effect modifiers for any of the five ethnic groups (see Appendix 1)?				
**Factor**	**Questions**	**Trial team answer**	**Proposed measures (presenting several options is acceptable)**	**Cost of measures**
**Biological**	What is the prevalence of the disease or condition in each ethnic group of the target population? Is this different from the prevalence in the general population?<br>Are there data that confirm that prevalence does not vary by ethnic group?			
	Is the severity of the disease different in each ethnic group? Is this different from the severity in the general population?			
	Other issues			
**Cultural**	To what extent are there cultural issues associated with one or more ethnic groups that might modify the intervention effect?			
	Other issues			
**Psychological**	[Or perhaps 'Cultural?]			
	Other issues			

2. Are there features of the intervention that make it hard for any of the five ethnic groups (see Appendix 1) to engage?*

Factor	Questions	Trial team answer	Proposed measures (presenting several options is acceptable)	Cost of measures
**What**	To what extent do the materials or information used or provided in the intervention limit the participation of some ethnic groups?			
	Other issues			
**Who**	To what extent does the person or persons delivering the intervention limit the participation of some ethnic groups?			
	Other issues			
**How**	To what extent does the mode of delivery (e.g. telephone, face-to-face, in groups) limit the participation of some ethnic groups?			
	Other issues			
**Where**	To what extent does where the intervention is delivered limit the participation of some ethnic groups?			
	Other issues			
**When and how much**	To what extent does the intensity of the intervention (e.g. the number of times it is delivered, over what time period, intensity, dose) limit the participation of some ethnic groups?			
	Other issues			

*These factors are taken from TIDieR (http://www.equator-network.org/reporting-guidelines/tidier/).

3. Are there features of the trial design and its delivery that make it hard for any of the five ethnic groups (or subgroups within them–see Appendix 1) to engage?				
**Factor**	**Questions**	**Trial team answer**	**Proposed measures (presenting several options is acceptable)**	**Cost of measures**
**Opportunity to participate**	To what extent are there eligibility criteria that are likely to exclude members of some ethnic groups for reasons other than their clinical eligibility for the trial (e.g. linguistic–'must speak English', location, gender, age, 'must have internet')			
	Other issues			
**Outcomes**	To what extent has the selection of trial outcomes, especially the primary outcome, involved representation across ethnic groups?			
	To what extent does how data are collected, where and by whom, limit the participation of some ethnic groups?			
	Other issues			
**Awareness of the trial**	To what extent does how (or who) potential participants are made aware of the trial limit the participation of some ethnic groups? Could staff attitudes limit the participation of some ethnic groups?			
	Other issues			

**Consent procedures**	To what extent does how trial information is delivered (or must be delivered in order to gain ethical approval) to potential participants (i.e. where, by whom, mode of delivery – written-only, verbal translations/multiple languages) limit the participation of some ethnic groups?			
	To what extent is the information you are providing to potential participants culturally relevant and appropriate (e.g. sensitive to cultural customs and beliefs, gender segregation, faith)?			
	Other issues			
