# Supplementary material for: Developing the INCLUDE Ethnicity Framework—a tool to help trialists design trials that better reflect the communities they serve
Source: Trials. 2021 May 10;22:337. doi: 10.1186/s13063-021-05276-8 (PMC8108025; doi:10.1186/s13063-021-05276-8)
Supplement: Supplementary file 6 — Additional file 6. [file 13063_2021_5276_MOESM6_ESM.pdf]

Ethnic minority groups are under-represented in many randomised trials– developing a practical tool to help trial designers avoid this in the future

4th February, Friends House, 173-177 Euston Road, London, NW1 2BJ

Main aim was to present a framework/checklist that Trial Forge and an NIHR INCLUDE subgroup had developed to help researchers to be more inclusive in terms of ethnicity (3-page table with example and some open space to comment).

General discussion points:

- Use the term underserved rather than underrepresented as “served” puts onus on the NIHR and other funders to serve the public as a provider of research evidence.
- We should be asking ‘For whom will the trial results be important?’ That leads us to explicitly think about our trial intention, who needs to be in our trial and what we need to do to ensure that this is the case. It requires a **cultural shift**, so that this approach is considered as central to a good trial as statistical support.
- Achieving this shift will need better engagement with the communities we intend to better serve.
- Easy to slip into thinking about the huge topic of how to better serve communities as a whole, but we need to start somewhere (today was about ethnicity), which can then serve as a template for other protected characteristics.
- A good place to start when thinking of expanding today’s work is to look through the prism of inequalities.
- Definitions of ethnic minority groups are difficult, but the five main groups used in the UK is probably the best we can do for now. We are interested in both biological reasons why treatment effects might be different but also cultural and social reasons.
- A lot going on in this area and constant mapping needed eg CLAHRC East Midlands Centre for BME Health toolkit (which involved a lot of good bottom-up work). The Trial Forge/NIHR INCLUDE approach is more aligned to a checklist framework that can then refer to the East Midlands explanatory/training document in a pyramid of information.

- One major handicap at present is complete absence of data on ethnicity in NIHR portfolio on which to base statements and measure changes due to well-meaning interventions – CRN could do more at local recruitment level and NIHR do more eg a new form of Table 1 in all trial reports including the final ethnicity breakdown of participants alongside a column of the ethnicity of the target population. That same Table 1 format could be used in other places, for example in submissions for ethical approval to show the proposed match between community and trial populations.
- Linked to data, we need some metrics against which we can measure success. Some baseline information about how things are now would be useful because this would enable a future assessment as to whether things have improved.
- Training around cultural competency for trial teams will go beyond the work described here. It is important and part of a bigger change needed to improve the way we do trials. It might help address some gatekeeping challenges among health professionals and others with whom we work to recruit to trials.
- A trial team's focus is usually preoccupied with overall sample size, rather than who is within that sample, which is sometimes more of a challenge. We need trial development groups to be equally concerned about the make-up of trial populations, not just the overall number.
- Agreed to avoid quota approach (i.e. you must include x% of individuals from a, b and c), which may perpetuate tokenism – at least for now unless the carrots don't work.
- For funders – be aware of additional costs – not so much with translations that could become easier to do with software (such as Reciteme https://reciteme.com/) with appropriate cultural checks, but with possible increase in sample sizes to account for different responses in different ethnic groups and even opposing treatment effects. Also, there are economies of scale and universities might invest more in such services given that it applies to so many studies. There is also likely to be a lot of overlap eg 80% of consent form texts are very similar, so there could be a central repository or store kept of generic but needed NIHR translation sections to avoid starting from scratch every time.

Trial Forge framework

- Trial Forge framework table is OK, but suggest it is used as a guidance document: the purpose of the work is to **make people think** and effect a behavioural cultural change as done successfully for PPI in NIHR research (and as we are now doing for research following patient need in terms of geography ie promoting recruitment for areas of the country with high disease burden where recruitment has been historically low). We need to avoid this becoming a tick box exercise responding only to the examples given rather than effecting culture change.

- The aim then is to to get trial teams to think more about the composition of their target and trial population at the design stage. So based on the Trial Forge template, have just four prompt questions with free text responses:

    1. Who should my trial apply to?
    2. Are the groups identified likely to respond in different ways?
    3. Will my study intervention make it harder for some groups to engage?
    4. Will the way I have designed the study make it harder for some groups to engage?

- We will need some exemplars but we don't want these to drive people's thought process or else they could end up as tick boxes. The above four questions are the top layer to promote thought; individuals who want more information can be signposted to more layers of information and detail in the Trial Forge guidance, the East Midlands guide (https://centreforbmehealth.org.uk/resources/toolkits/) and some exemplars.
- The examples and other resources can also be used for training trial staff, especially the East Midlands toolkit.
- We could have a PICO (Patient, Intervention, Comparator and Outcome) to orient thinking, probably before the four questions. Important to highlight 'To whom is this trial important?'
- How will judgements about groups and culture be made? How do we know these judgements are acceptable? Might need more than one person on a committee, might need a different group. Likely to influence how eg. funding panels are put together so that they better represent the community too. Could there be Equality champions? Who would be charged with this responsibility?
- Are prevalence (i.e. the number of people who have a particular condition or illness) and effect modifiers (i.e. things that change the effect a treatment has for a person) the same things, or different? It does seem possible to have two separate judgements for these rather than having them combined. Severity not the only thing too, maybe type or pattern as well.
- Can drop psychology from the framework but keep cultural as it is a broader term.
- The intervention part of the framework was fine, would be good if some examples were available. Acceptability is a tricky concept: varies from won't do at all, through to it's not really what I'd prefer but ok. How to make these judgements?
- Trial design and delivery needs to better reflect the trial journey a bit more.
- Could the TIDieR approach be applied to outcomes too (what, where etc).
- Also continue trial journey for intervention to analysis, dissemination and reporting.

- Use biological rather than genetic to describe how treatment effects might differ in different ethnic groups.
- Need to be aware that ethnic groups are not homogeneous and that not everyone within a particular group will have the same views, preferences, language skills etc. Again, need to think carefully about for whom the trial is important and judge accordingly. Need to reflect this in the table.
- For translation we might be able to get some help from institutions (i.e. a University might decide that it should be supporting language translation for populations in its immediate area), or each other. Not all documents need tailored translation and we could save effort and resource by sharing materials. We do need to be aware of cultural translation though, not just language.
- When we have a new version of the framework, we need to run some trials through it. A retrospective test using trials that had known problems recruiting people from diverse ethnic backgrounds would show us whether the framework would have identified those problems early.
- If there are resource implications, these need to be captured in the table.
- People using the table may need to make a decision as to which issue (age, sex, ethnicity etc) they want to focus on and then apply their thinking to that. There are many potential problems, which are the most important?
- Policymakers and journal editors are also key groups that we will, in due course, need to engage with.

Ideas for action (focused and sustained and not any old actions)

- Be clear what the purpose is (to better serve ethnic minorities in NHS research), the target audience (trial development groups mainly), the target behaviour (routine consideration of ethnicity in research) and what success looks like (which implies that it can be measured)
- Implement above 4 questions with leadership messages and nudges – eg NIHR programme director's message, text on application instructions.
- Perception out there that NIHR and other funders won't fund appropriate costs, which we need to overcome
- Financial nudges for the research community - think about a pilot £10k award as we have done for SWATs for additional work until the approach becomes normalised (which could take 5 years)
- Ask the key questions at Stage 1 standard application form with links to more information (but aware that wording for protected characteristics in general have already been developed)
- Think about ethnic diversity at all stages of research, but especially at the very applied end as in the NIHR HTA and Health Services and Delivery Research

Programmes. Some ethnicity feasibility issues can also be explored in feasibility studies

- Consider developing equality champions at funding committees - many PPI members are already embracing such a role given it is a legitimate public interest
- Increasing diversity in funding committees might also help
- Think about work across the entire research journey – applications and also in project monitoring and in editorial review.

2/3/2020
