# Supplementary material for: Developing the INCLUDE Ethnicity Framework—a tool to help trialists design trials that better reflect the communities they serve
Source: Trials. 2021 May 10;22:337. doi: 10.1186/s13063-021-05276-8 (PMC8108025; doi:10.1186/s13063-021-05276-8)
Supplement: Supplementary file 7 — Additional file 7. [file 13063_2021_5276_MOESM7_ESM.pdf]

Ensuring your trial is designed for all those in the community who could benefit

Trial name:	
**P**articipants:	
**I**ntervention:	
**C**omparator:	
**O**utcomes:	

1. **Who should my trial results apply to?**

2. **Are the groups of people identified in Q1 likely to respond to the treatment in different ways?**

3. **Will my trial intervention (or comparator) make it harder for any of the groups of people identified in Q1 to engage?**

4. **Will the way I have designed my trial make it harder for any of the groups of people identified in Q1 to engage with the trial?**

Appendix 1: worksheet for thinking through factors that might affect community-wide involvement

External factors that might change the effect of an intervention or its comparator for some groups		
**Biological**	How many people are estimated to have the disease or condition in each ethnic group in the target population?<br>Is this different from the frequency of the disease or condition in the general population?	Response:
	How does the severity of the disease vary between each ethnic group in the target population?<br>Is this different from the severity of the disease in the general population?	Response:
	How does the disease or condition present in people from each of the ethnic groups in the target population (this may include symptoms, type or pattern or rate of disease progression)?<br>Is this different from the presentation of the disease or condition in the general population?	Response:
	Other factors to consider	
**Cultural**	How might cultural practices, beliefs and traditions change the effect of the intervention in each of the ethnic groups in the target population?<br>Is this different from how cultural factors may change the effect of the intervention in the general population?	Response:
	Other factors to consider	

Intervention(s) factors that might affect how some groups engage*		
**What**	How involved were people from each of the ethnic groups in the target population in selecting or designing the trial intervention(s)?<br>Is this different from the level of involvement of people from the general population?	Response:
	Other factors to consider	
**Who**	How does the person or persons delivering the intervention limit initial and continued participation of people from each of the ethnic groups in the target population?<br>Is this different from how the person or persons delivering the intervention may limit initial and continued participation of the general population?	Response:
	Other factors to consider	
**How**	How does the mode of delivery (e.g. telephone, face-to-face, in groups) limit initial and continued participation of people from each of the ethnic groups in the target population?<br>Is this different from how the mode of delivery may limit initial and continued participation of the general population?	Response:
	Other factors to consider	
**Where**	How does where the intervention is delivered limit initial and continued participation of people from each of the ethnic groups in the target population?<br>Is this different from how where the intervention is delivered may limit initial and continued participation of the general population?	Response:

	Other factors to consider	
**When and how much**	How does the intensity of the intervention (e.g. the number of times it is delivered, over what time period, intensity, dose) limit initial and continued participation of people from each of the ethnic groups in the target population?<br>Is this different from how the intensity of the intervention may limit initial and continued participation of the general population?	Response:
	Other factors to consider	
*These factors are taken from TIDieR (http://www.equator-network.org/reporting-guidelines/tidier/).		

Trial outcome factors that might affect how some groups engage		
**What**	How involved were people from each of the ethnic groups in the target population in selecting the trial outcomes?<br>Is this different from the level of involvement of people from the general population?	Response:
	Other factors to consider	
**Who**	How does the person or persons who collect data limit initial and continued participation of each ethnic group in the target population?<br>Is this different from how the person or persons who collect data limit initial and continued participation of the general population?	Response:
	Other factors to consider	
**How**	How do data collection methods limit initial and continued participation of each ethnic group in the target population?<br>Is this different from how data collection methods limit initial and continued participation of the general population?	Response:
	Other factors to consider	
**Where**	How does where data are collected limit the initial and continued participation of each ethnic group in the target population?<br>Is this different from how where data are collected may limit initial and continued participation of the general population?	Response:
	Other factors to consider	

<table>
<tr><th colspan="3">Trial eligibility and participation factors that might affect how some groups engage</th></tr>
<tr><td rowspan="2">Eligibility</td><td>How are eligibility criteria likely to exclude members of each ethnic group in the target population for reasons other than their clinical eligibility for the trial (e.g. linguistic– ‘must speak English’, location, gender, age, ‘must have internet’)?
Is this different from how eligibility criteria are likely to exclude members of the general population?</td><td>Response:</td></tr>
<tr><td>Other factors to consider</td><td></td></tr>
<tr><td rowspan="4">Opportunity to participate</td><td>How does the way potential participants are made aware of the trial limit the initial participation of each ethnic group in the target population?
Is this different from how potential participants are made aware of the trial may limit initial participation of the general population?</td><td>Response:</td></tr>
<tr><td>How does the information that tells potential participants about the trial (e.g. participant information leaflet) limit the initial participation of each ethnic group in the target population?
Is this different from how the information that tells potential participants about the trial may limit initial participation of the general population?</td><td>Response:</td></tr>
<tr><td>How does the person who raises the trial with potential participants limit the initial participation of each ethnic group in the target population?
Is this different from how the person that tells potential participants about the trial may limit initial participation of the general population?</td><td>Response:</td></tr>
<tr><td>Other factors to consider</td><td></td></tr>
</table>

**Information and consent procedures**	How does the way trial information is delivered (or must be delivered in order to gain ethical approval) to potential participants (i.e. where, by whom, mode of delivery – written-only, verbal translations/multiple languages) limit initial participation of each ethnic group in the target population?<br>Is this different from how trial information is delivered may limit initial participation of the general population?	Response:
	How might cultural practices, beliefs and traditions change the way that information is perceived by each ethnic group in the target population?<br>Is this different from how cultural practices, beliefs and traditions change the way that information is perceived by the general population?	Response:
	Other factors to consider	

Factors that might affect the planned analysis of trial results		
**Retention**	How does participant retention vary across each ethnic group in the target population?	Response:
	Other factors to consider	
**Benefits**	How do the benefits of the trial intervention(s) differ between each ethnic group in the target population?	Response:
	Other factors to consider	
**Harms**	How do the harms of the trial intervention(s) differ between each ethnic group in the target population?	Response:
	Other factors to consider	
**Subgroup analyses**	How should variation in inclusion, benefits and harms between ethnic groups in the target population be explored? Should there be planned subgroup analyses?	Response:
	Other factors to consider	
**Interim analyses**	How should any interim analysis handle variation in inclusion, benefits and harms between ethnic groups in the target population?	Response:
	Other factors to consider	
**Stopping triggers**	How should any stopping rules handle variation in inclusion, benefits and harms between ethnic groups in the target population?	Response:
	Other factors to consider	

Factors that might affect the planned reporting and dissemination of trial results		
**What**	How involved were people from each of the ethnic groups in the target population in planning the reporting and dissemination of the trial results?<br>Is this different from the level of involvement of people from the general population?	Response:
	Other factors to consider	
**How**	How do planned reporting and dissemination methods limit engagement with each ethnic group in the target population?<br>Is this different from how reporting and dissemination methods limit engagement of the general population?	Response:
	Other factors to consider	
**Where**	How does where trial results will be reported and disseminated limit engagement of each ethnic group in the target population?<br>Is this different from where trial results are reported and disseminated limits engagement of the general population?	Response:
	Other factors to consider	

Appendix 2: Worksheet for thinking through measures to address factors that will prevent full community involvement

Factors that may prevent full community involvement	Proposed measures (several options may be needed)*	Cost of measures

*See https://centreforbmehealth.org.uk/resources/toolkits/ for suggestions for how to address factors that affect community-wide involvement.
