# Supplementary material for: Developing the INCLUDE Ethnicity Framework—a tool to help trialists design trials that better reflect the communities they serve
Source: Trials. 2021 May 10;22:337. doi: 10.1186/s13063-021-05276-8 (PMC8108025; doi:10.1186/s13063-021-05276-8)
Supplement: Supplementary file 8 — Additional file 8. [file 13063_2021_5276_MOESM8_ESM.pdf]

Ensuring your trial is designed for all who could benefit

The starting point for the design of all trials is to think about who will use the results. In other words, who is the trial for? The trial team then needs to do everything possible to make their results relevant to those identified as users of the results. This means thinking carefully about which parts of the community must be involved as participants.

The four questions below are intended to prompt trial teams to think about who needs to be involved in the trial, and how to make sure that their involvement is made possible. We believe these questions should always be considered by trial teams. Teams may also want to use the worksheets in Appendix 1 to help them think through their answers to the four questions.

1. **Who should my trial results apply to?**

   Who in the community could benefit from the intervention if it was found effective?

2. **Are the people identified in Q1 likely to respond to the intervention in different ways?**

   How might biology or culture mean that some groups in the community may respond differently to the intervention being evaluated?

3. **Will my trial intervention (or comparator) make it harder for any of the people identified in Q1 to engage?**

   How might the intervention itself (or comparator) and/or how they are delivered make it harder for some people to take part in the trial?

4. **Will the way I have designed my trial make it harder for any of the groups of people identified in Q1 to engage with the trial?**

   How might elements of trial design, such as eligibility criteria or the recruitment pathway, make it harder for some people to take part?

Appendix 1: worksheet for thinking through factors that might affect community-wide involvement

External factors that might change the effect of an intervention or its comparator for some groups		
**Biological**	How many people are estimated to have the disease or condition in each ethnic group in the target population?<br>Is this different from the frequency of the disease or condition in the general population?	Response:
	How does the severity of the disease vary between each ethnic group in the target population?<br>Is this different from the severity of the disease in the general population?	Response:
	How does the disease or condition present in people from each of the ethnic groups in the target population (this may include symptoms, type or pattern or rate of disease progression)?<br>Is this different from the presentation of the disease or condition in the general population?	Response:
	Other factors to consider	
**Cultural**	How do people in each ethnic group seek healthcare for this disease or condition at the same rate?<br>How might cultural practices, beliefs and traditions change the effect of the intervention in each of the ethnic groups in the target population?<br>Is this different from how cultural factors may change the effect of the intervention in the general population?	Response:
	Other factors to consider	

Intervention(s) factors that might affect how some groups engage*		
**What**	How involved were people from each of the ethnic groups in the target population in selecting or designing the trial intervention(s)?<br>Is this different from the level of involvement of people from the general population?	Response:
	Other factors to consider	
**Who**	How does the person or persons delivering the intervention limit initial and continued participation of people from each of the ethnic groups in the target population?<br>Is this different from how the person or persons delivering the intervention may limit initial and continued participation of the general population?	Response:
	Other factors to consider	
**How**	How does the mode of delivery (e.g. telephone, face-to-face, in groups) limit initial and continued participation of people from each of the ethnic groups in the target population?<br>Is this different from how the mode of delivery may limit initial and continued participation of the general population?	Response:
	Other factors to consider	
**Where**	How does where the intervention is delivered limit initial and continued participation of people from each of the ethnic groups in the target population?<br>Is this different from how where the intervention is delivered may limit initial and continued participation of the general population?	Response:

	Other factors to consider	
**When and how much**	How does the intensity of the intervention (e.g. the number of times it is delivered, over what time period, intensity, dose) limit initial and continued participation of people from each of the ethnic groups in the target population?<br>Is this different from how the intensity of the intervention may limit initial and continued participation of the general population?	Response:
	Other factors to consider	
*These factors are taken from TIDieR (http://www.equator-network.org/reporting-guidelines/tidier/).		

Trial outcome factors that might affect how some groups engage		
**What**	How involved were people from each of the ethnic groups in the target population in selecting the trial outcomes?<br>Is this different from the level of involvement of people from the general population?	Response:
	Other factors to consider	
**Who**	How does the person or persons who collect data limit initial and continued participation of each ethnic group in the target population?<br>Is this different from how the person or persons who collect data limit initial and continued participation of the general population?	Response:
	Other factors to consider	
**How**	How do data collection methods limit initial and continued participation of each ethnic group in the target population?<br>Is this different from how data collection methods limit initial and continued participation of the general population?	Response:
	Other factors to consider	
**Where**	How does where data are collected limit the initial and continued participation of each ethnic group in the target population?<br>Is this different from how where data are collected may limit initial and continued participation of the general population?	Response:
	Other factors to consider	

Trial eligibility and participation factors that might affect how some groups engage		
**Eligibility**	How are eligibility criteria likely to exclude members of each ethnic group in the target population for reasons other than their clinical eligibility for the trial (e.g. linguistic– 'must speak English', location, gender, age, 'must have internet')?<br>Is this different from how eligibility criteria are likely to exclude members of the general population?	Response:
	Other factors to consider	
**Opportunity to participate**	How does the way potential participants are made aware of the trial limit the participation of each ethnic group in the target population?<br>Is this different from how potential participants are made aware of the trial?	Response:
	How does the information that tells potential participants about the trial (e.g. participant information leaflet) limit the participation of each ethnic group in the target population?<br>Is this different from how the information that tells potential participants about the trial may limit initial participation of the general population?	Response:
	How does the person who raises the trial with potential participants limit the participation of each ethnic group in the target population?<br>Is this different from how the person that tells potential participants about the trial may limit initial participation of the general population?	Response:
	Other factors to consider	

**Consent procedures**	How does the way consent is taken (i.e. where, by whom, written vs verbal, verbal translations/multiple language support) limit participation of each ethnic group in the target population?<br>How does the way people would like to interact with family and healthcare workers before providing consent differ by each ethnic group in the target population?<br>How does the way the research team can check how consent information is understood differ for each ethnic group in the target population?<br>Is this different from how trial information is delivered may limit initial participation of the general population?	Response:
	How might cultural practices, beliefs and traditions change the way that information is perceived by each ethnic group in the target population?<br>Is this different from how cultural practices, beliefs and traditions change the way that information is perceived by the general population?	Response:
	Other factors to consider	

Factors that might affect the planned analysis of trial results		
**Retention**	How does participant retention vary across each ethnic group in the target population?	Response:
	Other factors to consider	
**Benefits**	How do the benefits of the trial intervention(s) differ between each ethnic group in the target population?	Response:
	Other factors to consider	
**Harms**	How do the harms of the trial intervention(s) differ between each ethnic group in the target population?	Response:
	Other factors to consider	
**Subgroup analyses**	How should variation in inclusion, benefits and harms between ethnic groups in the target population be explored? Should there be planned subgroup analyses?	Response:
	Other factors to consider	
**Interim analyses**	How should any interim analysis handle variation in inclusion, benefits and harms between ethnic groups in the target population?	Response:
	Other factors to consider	
**Stopping triggers**	How should any stopping rules handle variation in inclusion, benefits and harms between ethnic groups in the target population?	Response:
	Other factors to consider	

Factors that might affect the planned reporting and dissemination of trial results		
**What**	How involved were people from each of the ethnic groups in the target population in planning the reporting and dissemination of the trial results?<br>Is this different from the level of involvement of people from the general population?	Response:
	Other factors to consider	
**How**	How do planned reporting and dissemination methods limit engagement with each ethnic group in the target population?<br>Is this different from how reporting and dissemination methods limit engagement of the general population?	Response:
	Other factors to consider	
**Where**	How does where trial results will be reported and disseminated limit engagement of each ethnic group in the target population?<br>Is this different from where trial results are reported and disseminated limits engagement of the general population?	Response:
	Other factors to consider	

Appendix 2: Worksheet for thinking through measures to address factors that will prevent full community involvement

Factors that may prevent full community involvement	Proposed measures (several options may be needed)*	Cost of measures

*See https://centreforbmehealth.org.uk/resources/toolkits/ for suggestions for how to address factors that affect community-wide involvement.
