# Supplementary material for: Developing the INCLUDE Ethnicity Framework—a tool to help trialists design trials that better reflect the communities they serve
Source: Trials. 2021 May 10;22:337. doi: 10.1186/s13063-021-05276-8 (PMC8108025; doi:10.1186/s13063-021-05276-8)
Supplement: Supplementary file 9 — Additional file 9. [file 13063_2021_5276_MOESM9_ESM.zip › renamedR1_70428.pdf]

Ensuring your trial is designed for all who could benefit

Key questions

The starting point for all trials is to think about who the trial is for. The trial team then needs to do everything possible to make their trial relevant to the people to whom the results are intended to apply (often patients) and those expected to apply them (often healthcare professionals). This means thinking carefully during trial planning and design about who should be involved as participants.

The four questions below are intended to prompt trial teams to think about who should be involved as participants, and how to facilitate their involvement as much as possible. These questions should always be considered by trial teams in partnership with patient and public partners, including individuals from, or representing, groups identified in Question 1.

We recommend that trial teams use the worksheets to help them think through their answers to the four key questions.

Question 1–

Who should my trial results apply to?

Which groups in the community could benefit from the intervention if it was found effective, or benefit from not having it if it was found ineffective and/or harmful?

Question 2–

Are the groups identified in Question 1 likely to respond to the treatment in different ways?

How might the disease or cultural factors mean that some groups in the community respond to, or engage with, the treatment(s) being tested in different ways?

Question 3–

Will my trial intervention and/or comparator make it harder for any of the groups identified in Question 1 to engage with the intervention and/or comparator?

How might the intervention and/or comparator, including how they are provided, make it harder for some groups in the community to take part in the trial?

Question 4–

Will the way I have planned and designed my trial make it harder for any of the groups identified in Question 1 to consider taking part?

How might elements of trial design, such as eligibility criteria or the recruitment and consent process, make it harder for some groups in the community to take part?
